# Curcumin Microcapsule Formulations for Prolong Persistence in the Photodynamic Inactivation of *Aedes aegypti* Larvae

**DOI:** 10.3390/pharmaceutics17040496

**Published:** 2025-04-09

**Authors:** Matheus Garbuio, Larissa Marila de Souza, Lucas Danilo Dias, Jean Carlos Ferreira Machado, Natalia Mayumi Inada, Hernane da Silva Barud, Edgar Aparecido Sanches, Francisco Eduardo Gontijo Guimarães, Ana Paula da Silva, Alessandra Ramos Lima, Vanderlei Salvador Bagnato

**Affiliations:** 1 São Carlos Institute of Physics (IFSC), University of São Paulo (USP), São Carlos 13566-590, SP, Brazil; matthew.gohan@gmail.com (M.G.); larissamarila@hotmail.com (L.M.d.S.); nataliainada@ifsc.usp.br (N.M.I.); guimarae@ifsc.usp.br (F.E.G.G.); paulalsir@gmail.com (A.P.d.S.); ramos.alessandra.09@gmail.com (A.R.L.); vander@ifsc.usp.br (V.S.B.); 2 Environmental Biophotonics Laboratory, São Carlos Institute of Physics, University of São Paulo, São Carlos 13566-590, SP, Brazil; 3 PPG Biotec, Federal University of São Carlos, São Carlos 13565-905, SP, Brazil; 4 Laboratório de Novos Materiais, Universidade Evangélica de Goiás (UniEvangélica), Anápolis 75083-515, GO, Brazil; 5 Biopolymers and Biomaterials Laboratory (BioPolMat), University of Araraquara—UNIARA, Araraquara 14801-320, SP, Brazil; hernane.baruud@gmail.com (J.C.F.M.); hernane.barud@gmail.com (H.d.S.B.); 6 Laboratory of Nanostructured Polymers (NANOPOL), Federal University of Amazonas (UFAM), Manaus 69080-005, AM, Brazil; sanchesufam@ufam.edu.br; 7 Department of Biomedical Engineering, Texas A&M University, College Station, TX 77843, USA

**Keywords:** photodynamic inactivation, curcumin microencapsulation, vector control, photoinsecticide, spray drying

## Abstract

**Background:** Viral diseases including dengue, zika, chikungunya, and yellow fever remain a significant public health challenge, primarily due to the increasing resistance of these vectors, the *Aedes aegypti* mosquito, to conventional control methods. **Objectives:** Herein, a microencapsulated curcumin formulation was developed and characterized using spray-drying technology, with D-mannitol and starch as encapsulating agents. After microencapsulation, photolarvicidal tablet formulations (Formulated Curcumin Tablets—FCT) were prepared, varying the proportions of starch and pectin: FCT1 (60% starch), FCT2 (35% pectin and 25% starch), and FCT3 (42.5% pectin and 17.5% starch), while maintaining 10% curcumin and 30% D-mannitol in all formulations. The main goal was to enhance the stability and efficacy of curcumin as a photolarvicidal agent. **Methods:** The formulation was characterized by UV-Vis spectroscopy, confocal microscopy, thermal analysis (TG and DSC), scanning electron microscopy (SEM), fourier transform infrared spectroscopy (FTIR), and photodegradation assays under fluorescent light. **Results:** The photodynamic inactivation (PDI) of *Ae. aegypti* larvae was evaluated under white, fluorescent light exposure, and the formulation exhibited a significantly enhanced larvicidal activity compared to free curcumin, with a 57-fold reduction in LC_50_ (LC_50-24h_ = 0.27 mg/L). Additionally, the most effective formulation, FCT2, maintained its residual activity for 27 days, reinforcing that curcumin microencapsulation, combined with PDI, can extend vector control. Release studies under different pH conditions confirmed a controlled release mechanism, favoring environmental stability. **Conclusions:** The results indicate that microencapsulated curcumin has great potential as a sustainable photoinsecticidal agent, offering stability, efficacy, and a promising alternative for managing *Ae. aegypti* larval populations.

## 1. Introduction

Dengue, Zika, and chikungunya are arboviral diseases mainly found in South/Central America, Asia, and Africa [1]. However, the increasing globalization has accelerated the spread of these viral diseases [2]. These infections are transmitted by the bites of infected female *Aedes aegypti* mosquitoes, and cases of resistance have been described [3]. In 2019, over 2.7 million cases of dengue were reported, including 22,127 severe cases and 1206 deaths [4]. Additionally, the World Health Organization (WHO) estimates about 50 million dengue infections annually, leading to 22,000 deaths, mostly children [5].

Some strategies have been reported to combat these viral infections such as vector control (using elimination of breeding sites, use of insecticides, biological control), vaccination, and community Engagement and Education [6,7]. However, efforts have been hampered by the development of resistance in mosquitoes and larvae, as well as the environmental impact caused by insecticides affecting non-target species. In this regard, new and effective tools (e.g., antimicrobial photodynamic therapy—aPDT) must be developed and studied aiming to overcome these challenges [8].

aPDT is characterized by the combined action between a photosensitizing molecule (PS), molecular oxygen (O_2_), and a light source to produce reactive oxidative species (ROS) followed the oxidation reaction of target biomolecules present protozoa, virus, bacteria, fungi and larvae [9,10,11]. Among the PS report for larval photoinactivation [12], some examples include riboflavin [13], methylene blue, rose bengal [14] and curcumin [15,16,17].

Curcumin is a polyphenolic compound known for its complex structure, capable of existing in different tautomeric forms, predominantly the keto and enol forms. These structural variations influence its physicochemical behavior, including solubility, stability, and biological activity in aqueous environments [18]. Highlighting the PS, the natural-based curcumin was first applied showing as a promising photoactive molecule against *Ae. aegypti* larvae. Then, aiming to improve its performance, PS was formulated with mannitol [19] and sucrose [20] in order to increase the concentration and the water solubility for environmental application. However, its stability, efficiency, and dissolution profile in aqueous medium should be improved.

In this study, synthetic curcumin was used due to its higher purity, reproducibility, and structural consistency [21,22]. Although the structural complexity of curcumin, such as the keto and enolic tautomeric forms, is well recognized [23,24,25], this work did not aim to investigate these forms individually. The encapsulation strategy was used to protect the photoactive structure and improve physicochemical stability and applicability as a potential photolarvicidal agent [19,26,27].

This work aimed to develop a pharmaceutical formulation of curcumin containing mannitol and pectin by using a spry dryer technology. Due to curcumin’s structural complexity, particularly its ability to exist in tautomeric forms, the design of a stable formulation must consider the preservation of these forms. These excipients were chosen to increase the solubility and stability of curcumin as well as to provide a controlled release profile for environmental applications.

This formulation was fully characterized by UV-Vis, infrared, TG analysis, confocal microscopy, DSC analysis, scanning electron microscopy. Moreover, the developed formulation was evaluated for photodegradation when exposed to fluorescent light (white light), proving its efficacy as a photolarvicide in bioassays with *Ae. aegypti* larvae.

## 2. Materials and Methods

### 2.1. Spray-Drying Microencapsulation Process

**Figure 1:**
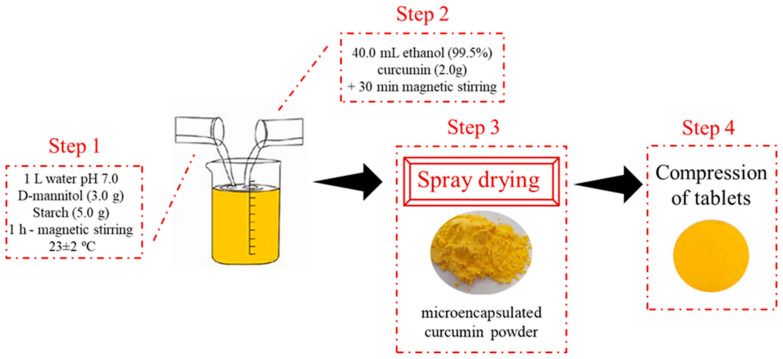
Method for producing microencapsulated curcumin formulations. Step 1: homogenization of starch and D-mannitol in water; Step 2: addition of curcumin in 99.5% ethanol; Step 3: drying the solution by spray-drying technique; Step 4: compression of the microcapsules into tablets.

The wall materials, D-mannitol ≥ 98% (3.0 g) (Sigma-Aldrich, St. Louis, MO, USA), curcumin 100% (PDT Pharma-LTDA, Cravinhos, SP, Brazil) and soluble starch ≤98% (5.0 g) Exôdo Científica (Sumaré, SP, Brazil) were used. The protocols for obtaining microencapsulated curcumin were based on the literature with modifications [28]. Figure 1 to explain the formulation methods. The biopolymers were dissolved in 1 L of water adjusted to pH 7.0 and kept under constant magnetic stirring at 23 ± 2 °C for 1 h (Step 1). Simultaneously, 2.0 g of curcumin powder, purchased from a commercial supplier, was dissolved in 40.0 mL of 99.5% ethanol, stirred for 1 h, and protected from light. The prepared solutions were then combined and stirred for an additional 30 min, also protected from light, to ensure complete homogenization (Step 2).

The liquid mixture was dried by spray drying using a spray dryer (model MsDi 1.0, LabMaq do Brasil Ltd., Ribeirão Preto, Brazil). (a) under the following operational conditions: inlet temperature of 130–140 °C and outlet temperature of 70–80 °C; drying air flow rate of 1.20 m^3^/min; compressed air flow rate of 0.3 L/h; compressed air pressure of 6 bar; and atomizer nozzle diameter of 1.22 mm, and compressed air consumption 45 L/h. The microencapsulated curcumin powder was protected from light and stored at 4 °C (Step 3).

### 2.2. Formulations Curcumin Tablets (FCT)

**Figure 2:**
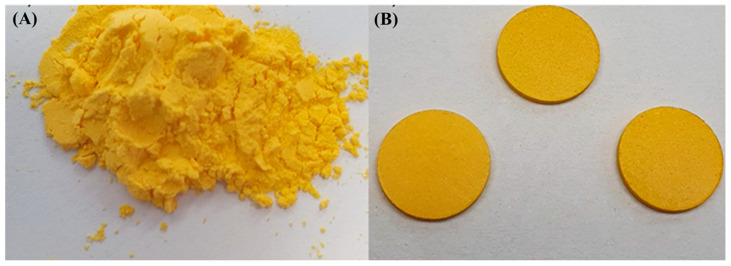
(**A**) microencapsulated curcumin obtained without spray drying. (**B**) FCT obtained via press with a force of 4 kN.

The tablets (Figure 2) were obtained by physically mixing the powder resulting from the spray microencapsulated curcumin and the excipients D-mannitol, pectin and starch, in the final proportions described in Table 1. The content of the curcumin fraction and D-mannitol in the tablets were fixed at 10% and 30% (*w*/*w*), respectively, varying the fractions of the excipient’s pectin and starch.

Mixing the formulation (Figure 2A) to obtain the tablets was performed manually with the aid of a mortar and pestle (Figure 2B). The compression of the active curcumin encapsulated with the mixture of excipients was carried out using a tablet with a diameter of 12 mm in a hydraulic press with a force of 4 kN, for 10 s, to obtain tablets of similar hardness. Placebo tablets were also produced, without the addition of curcumin (Figure 1—Step 4).

### 2.3. Release Efficiency of Microencapsulated Curcumin and Formulation

The release profile of curcumin from the microcapsules was monitored over time in an aqueous medium. Curcumin quantification was performed using ultraviolet-visible (UV-Vis) absorption spectroscopy [27]. Aliquots of 5.0 mL were removed at pre-determined time intervals, followed by replacement of 5.0 mL of said pH. Aliquots of 5.0 mL were removed at 10 min time intervals, followed by replacement of 5.0 mL. Curcumin present in the aliquot was then extracted by adding 5.0 mL of 99% ethyl alcohol (EtOH), stirring for 20 min, protected from light. The solution was then filtered using black band quantitative filter paper (µm) and analyzed using a UV-Vis spectrophotometer at 430 nm. A calibration curve (R = 0.9999) was established (SM) with concentrations ranging from 0.0 to 7.0 mg/L in ethanol. Curcumin extraction from the microcapsules was carried out using a solvent extraction method (SM). The encapsulation efficiency (*EE*%) was calculated based on Equation (1):

(1) $$EE\%:\frac{MCA-RCE\;}{QCA}\times 100$$

where—*MCA*: Microencapsulated curcumin dispersed in water; *RCE*: Released curcumin extracted from ethyl alcohol after being released from microcapsules; *QCA*: Total quantity of curcumin added.

The controlled release study of curcumin from the tablet formulation was carried out under static conditions, without stirring. The controlled release was carried out in triplicate at a temperature of 25 °C, in static conditions for the tablet formulation that showed the greatest photolarvicidal effect. Curcumin release formulated FCT1, FCT2 e FCT3 from the tablet was carried out at pH 3.0; 7.0 and 11.00, in triplicate. The tablets were added inside a 200-mesh filter bag (height: 8.50 cm/Width: 10.00 cm), suspended by Platinum Carbon Hybrid Fluoro Monofilament (0.40 mm) in 1000 mL. The quantification of curcumin released as a function of time was determined using a previously determined displacement curve, available in Supplementary Materials (SM). All data were processed and analyzed using Origin 2020 software (OriginLab^®^, version 9.7.0, USA).

Formulation FCT2 showed that the release of the active ingredient was slower depending on the pH. The release of FCT2 was explored in a mathematical model that shows how the release of the active ingredient behaves depending on the pH, which is relevant for understanding the dispersion of curcumin in the microbiota in *Ae. aegypti* breeding sites, since there is a variation in pH in the environment. After the release test, the values obtained from the analyzes were plotted and adjusted to the Korsmeyer–Peppas model according to Equation (2).

(2) $$\frac{Mt}{\;M_{\infty }}=Kt^{n}$$

where *K* is the diffusion constant, *n* is the release exponent that characterizes the active release mechanism, *Mt* is the amount of active released in time *t*, *M* the total amount of active agent in an infinite time, and $\frac{Mt}{M\infty }$ is the fraction of the active agent released over time *t* [29,30,31].

### 2.4. Characterization of Curcumin Microcapsule and Formulations

Scanning electron Microscopy—The morphological characteristics of the free curcumin, starch, D-mannitol and curcumin microcapsules were analyzed by high-resolution scanning electron microscopy (SEM-FEG), with a field emission electron gun (SEM-FEG, Supra 35-VP, Carl Zeiss, Jena, Germany). The samples were fixed to a substrate on carbon tape, coated with gold/palladium and analyzed at 2.5 kV. Particle size measurement was performed using ImageJ software (version 1.50i). For the size distribution of curcumin microcapsules, it was considered that they had a spherical shape. Size measurements of more than 300 particles the starch and curcumin microcapsules found in arbitrarily chosen regions of the grid were obtained. The histograms were adjusted considering normal distribution.

Confocal microscopy microencapsulated curcumin—The morphology of microencapsulated curcumin was imaged using a confocal fluorescence microscope (Zeiss-LSM780, Zeiss, Jena, Germany) with a Coherent Chameleon laser (Ti:Sapphire) as a two-photon (2P) excitation source tuned to a wavelength of 800 nm with a laser pulse of 80 MHz. The images of microencapsulated curcumin were obtained by fixing the excitation fluorescence at intervals of 450–500 nm (channel 1) and 500–700 nm (channel 2).

Thermogravimetry (TG) and Derivative TG (DTG)—The thermal behavior of samples was also analyzed via TG and derivative TG using a simultaneous thermal analysis module—SDT equipment (Q 600, TA Instruments, New Castle, DE, USA). The analyses were performed in an inert N_2_ atmosphere, with a flow rate of 50 mL/min, temperature range of 10–650 °C, heating rate of 10 °C/min and sample mass of 10 mg in an aluminum crucible.

Differential scanning calorimetry (DSC) The thermal transitions of the samples were obtained with DSC using (TA Instruments SDT-Q600, New Castle, DE, USA). The equipment operating parameters were an inert atmosphere of N_2_ with flow of 50 mL/min, sample mass around 10 mg in an aluminum crucible and temperature range of 10–650 °C with heating rates of 10 °C/min.

Attenuated Total Reflectance Fourier Transform Infrared Spectroscopy (ATR-FTIR)—Mid-infrared spectra were obtained in the region of 650–4000 cm^−1^ with a spectral resolution of 16 cm^−1^ on an Agilent Cary 630 FTIR spectrometer (Agilent Technologies, Santa Clara, CA, USA).

### 2.5. Photodegradation Assay of Microencapsulated Curcumin Formulations

The photodegradation of formulations FCT1, FCT2, and FCT3 was carried out by grinding one tablet of each formulation in an agate mortar, followed by adding the resulting powder to glass containers containing 1 L of distilled water. The samples were exposed to fluorescent lamp irradiation (white light) at an intensity of 0.83 mW/cm^2^ for 30 days, in triplicate. Aliquots were collected from the containers at 0, 5, 10, 15, 20, 25, and 30 days, corresponding to continuous exposure to irradiation and a photoperiod of 12 h/12 h. Curcumin extraction from the samples was performed as described in Section 2.3, and analyses were conducted using UV-Vis absorption spectroscopy (Cary Bio^®^50—Santa Clara, CA, USA) in the range of 200 to 800 nm, employing a quartz cuvette with two polished faces and a 10 mm optical path. The degraded curcumin concentration was determined through the SM calibration curve.

## 3. Photodynamic Inactivation of *Aedes aegypti* Larvae

Rockerfeller mosquito eggs were kindly donated by the Laboratory of Physiology and Control of Arthropod Vectors at Instituto Oswaldo Cruz, Rio de Janeiro, RJ, Brazil (LAFICAVE—IOC/Fiocruz). The bioassays were conducted at the Environmental Biophotonics Laboratory of the Optics Group, São Carlos Institute of Physics, University of São Paulo (IFSC-USP). The ribbons with the eggs were placed in plastic trays containing 2 L of dechlorinated water, in an air-conditioned room at 30 °C with a photoperiod (12:12 h light/dark) and humidity of 60.0 ± 5%. After hatching, the larvae were fed with Alcon BASIC^®^ MEP 200 Complex food (Alcon, Santa Catarina, Brazil) until they reached the 3rd stage of development, to carry out photodynamic inactivation tests.

Larvicidal activity was evaluated using the World Health Organization protocol [32], with adaptations. In total, 25 larvae were added to plastic containers, with a volume of 50 mL. Concentrations of curcumin microcapsules and the three curcumin formulations (FCT1, FCT2 and FCT3) were tested: 0.01, 0.04, 0.07, 0.15, 0.31, 0.62, 1.25, and 2.5 mg/L. In addition, two control groups were carried out: light control and dark control. And for free curcumin the concentrations were: 0.97, 1.95, 3.9, 7.81, 15.6, 31.25, 62.5, and 125 mg/L.

The experiments were carried out in triplicate on independent days, irradiated with a system of 18 W white, fluorescent lamps, irradiance of 0.83 mW/cm^2^, light dose 35.86 J/cm^2^. Larval mortality was verified 24 h after the start of the tests. From the mortality rates of lethal concentrations (LC_50-24h_), a dose–response curve was calculated using non-linear regression in Origin 2020 software (OriginLab^®^, version 9.7.0, USA).

### 3.1. Residual Effect of Curcumin Microcapsule Formulations on Ae. aegypti Larvae

The residual activity of the tablet formulations FCT1, FCT2, and FCT3 was evaluated in the laboratory according to the methodology described in Vani et al. 2018, with modifications [33]. The tablets were added to plastic containers containing 1 L of distilled water, to which groups of 25–30 third instar (L3) *Ae. aegypti* larvae were introduced. Tablets without curcumin microcapsules were used as negative controls. Larval mortality was checked daily, and all larvae (dead or alive) were removed from the container and replaced with new larvae. This replacement continued until the tablets completely lost efficacy over 30 days. The experiments were conducted in triplicate under irradiation from a system of 18 W white, fluorescent lamps, with an irradiance of 0.83 mW/cm^2^ and a photoperiod of 12 h/12 h.

### 3.2. Confocal Laser Scanning Fluorescence Microscopy of Aedes aegypti Larvae

The third stage larvae (L3) were exposed to the formulations for 20 min and then washed with distilled water and placed on slides for triplicate analysis or triplicate analysis [15]. The distribution of FCT1, FCT2, and FCT3 in *Ae. aegypti* was determined using inverted confocal fluorescence microscopy (Zeiss LSM780, Jena, Germany), equipped with a Chameleon laser (Ti, Coherent, Santa Clara, CA, USA) as a two-photon (2P) excitation source at 800 nm, and a Plan-Apochromat objective lens (20×). The images were obtained in channel mode, considering the spectral regions of larval autofluorescence and curcumin emission separately. Therefore, the images of curcumin formulations in larvae were obtained by setting the fluorescence regions to 420–700 nm (channel 1) and 500–700 nm (channel 2). Each pixel of the image contains fluorescence intensity from the two spectral regions separately. Images were collected using appropriate optical filters to exclude laser light.

## 4. Results and Discussions

### 4.1. Spray Dryer Yield

The yield of microencapsulated curcumin was 43 ± 3%, with encapsulating agents based on starch and D-mannitol biopolymers. Although the conveyors used in this study did not obtain a yield greater than 50% of the reference value for processes considered to have good yield, the result obtained was significant, especially considering the use of laboratory-scale equipment [28]. It is worth highlighting that the sample yield is highly influenced by the operating conditions of the equipment, the type of feed (emulsion or solution) and the volume processed [34].

The yield was similar to that obtained for microencapsulated curcumin by spraying. Mixtures of Arabic gum, maltodextrin and starch were used, which obtained a yield of 48%, even though the encapsulating biopolymers were different [35]. Other studies also showed similar yield values using the spray-drying technique, which encapsulated curcumin with the biopolymers Arabic gum and chitosan [28,36].

### 4.2. Release Efficiency of Microencapsulated Curcumin

**Figure 3:**
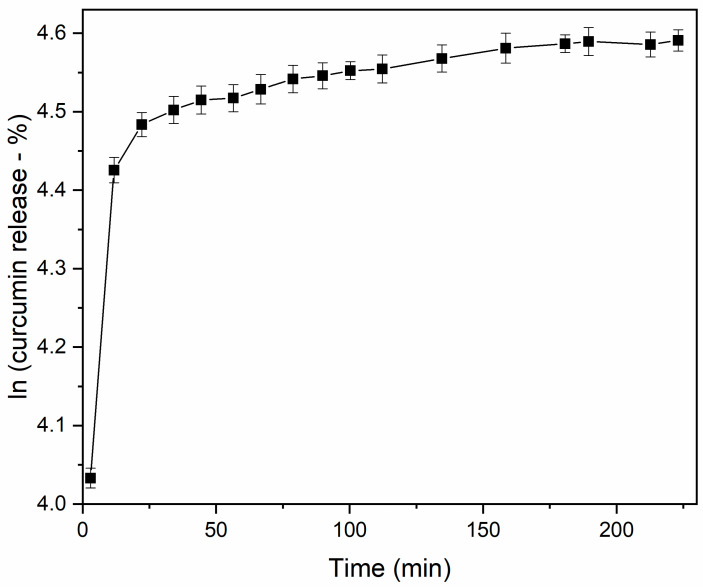
Release profile of curcumin from spray-dried process. The Y-axis represents the natural logarithm (*ln*) of the concentration (%) of curcumin released over time.

Figure 3 shows the release profile of the microcapsules obtained by the spray-drying process. Analyzing the curve, we observed that the microcapsules follow the biphasic release kinetics (it presents a curve with an initial peak followed by a more stable and prolonged release phase reaching a saturation of the medium by diffusion), presenting a sudden increase in the release of curcumin in the first 15 min (approximately 80%) followed by a slow release [37].

In this case, the rapid release behavior of curcumin is known as “explosive release” in this phase. The release happens through processes such as controlled diffusion, degradation of the encapsulating polymer, or erosion of the matrix that surrounds the active. In this work, the explosion effect is initially due to the highly hydrophilic characteristic of the starch carrier [38]. As soon as the microcapsules came into contact with the working solution, the starch underwent rapid swelling, and the curcumin particles diffused almost immediately out of the microcapsules [39].

The burst effect can be highly desirable in certain situations. Rapid release or high initial delivery rates may be desirable in situations involving the elimination of vector larvae, since the immediate delivery of a part of the curcumin load would ensure the total or partial elimination of the organisms existing in the breeding facility. A long-term controlled release system is not desirable due to the rapid development of the aquatic stage of *Ae. aegypti* (average of 5–6 days at 28 °C) [40].

The second phase of biphasic release involves a slower and more sustained release [37], maintaining a relatively constant concentration of the active substance in the medium for an extended duration, as observed after 16–150 min and after that a moment of stabilization and saturation with the medium. Due to this biphasic nature, the release kinetics do not follow a simple exponential. Instead, the release profile can be best described by an equation $ln\left(Rel\right)=A_{0}+\frac{t}{\tau }$ showing the release constant changes the time as the release phase changes.

### 4.3. Scanning Electron Microscopy

**Figure 4:**
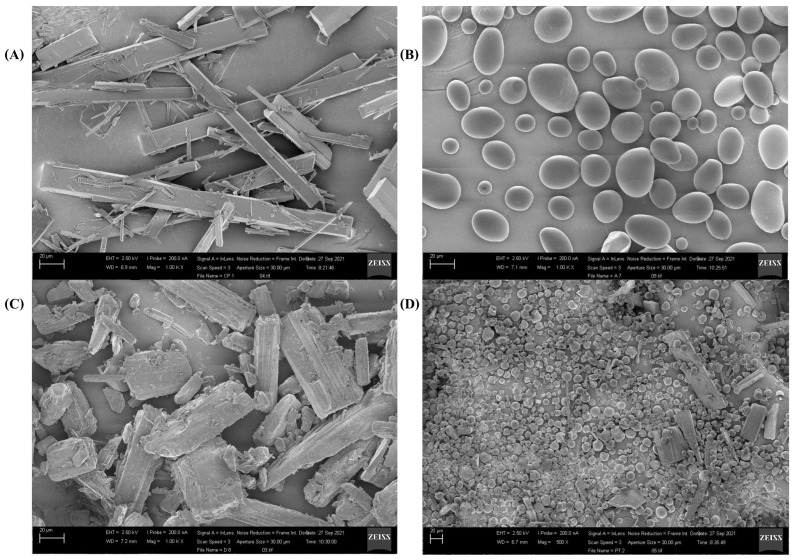
FEG-SEM images of morphology scale 20 µm: (**A**) active curcumin, (**B**) starch, (**C**) D-mannitol, and (**D**) microencapsulated curcumin.

Figure 4 shows the surface morphology of the active curcumin, excipients starch and D-mannitol, and the microencapsulated curcumin. Each material exhibited distinct morphological characteristics. The free curcumin (Figure 4A) exhibits the form of well-faceted crystals. Starch (Figure 4B) has a spherical and regular morphology, with a smooth surface. D-mannitol, (Figure 4C), is characterized by an irregularly shaped rod structure. Finally, Figure 4D of microencapsulated curcumin shows that the spherical morphology is not homogeneous with surface deformations of the microcapsules being smooth and rough. It is noted that the curcumin microcapsules indicated by arrows in the image may be hollow inside. Similar morphologies for curcumin microencapsulated by spray-drying method have been reported in different biopolymers [28,35,37].

**Figure 5:**
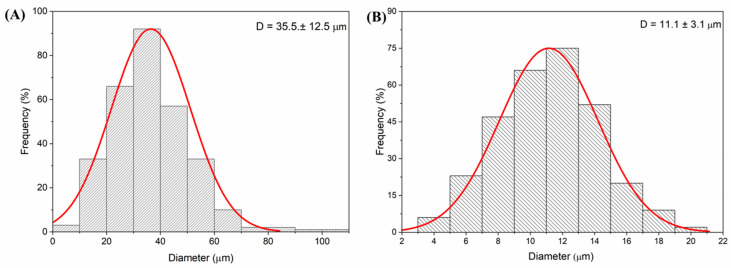
Microparticle size distribution: (**A**) starch—average size of 35 ± 12.5 µm and (**B**) microencapsulated curcumin—average size of 11.1 ± 3.1 µm.

The morphology of the starch microparticle alone and microencapsulated curcumin are similar, but the average size distribution is different from Figure 5. The histograms were fitted considering a normal distribution. This difference can be attributed to the spray-drying process. The distribution in Figure 5A shows that the starch microparticles have an average diameter of around 35 ± 12.5 µm. And Figure 5B presents the distribution for microencapsulated curcumin microparticles with an average size of about 11.1 ± 3.1 µm.

The average size of curcumin microencapsulated in starch and D-mannitol was similar to that obtained by Cano-Higuita and collaborators [35] who demonstrated for curcumin microencapsulated in different biopolymers via spray drying, maltodextrin 75% and modified starch 25% obtained an average size of 21.05 ± 1.10 μm, while for a mixture in equal proportions of the gum excipients arabica, maltodextrin, and modified starch was 12.57 ± 0.44 μm [35].

### 4.4. Microcapsules Morphology by Confocal Microscopy

**Figure 6:**
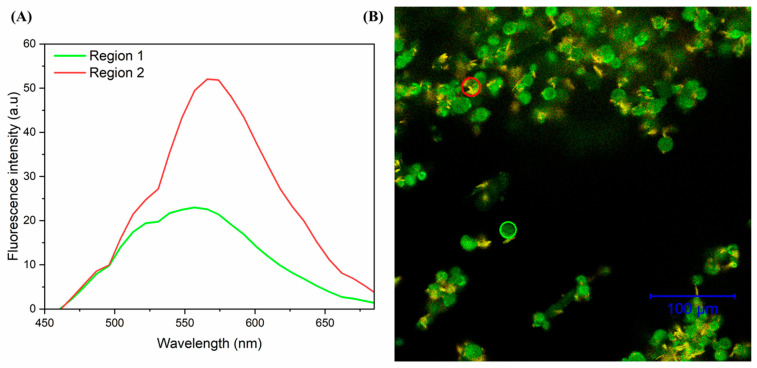
Confocal microscopy of microencapsulated curcumin. (**A**) Fluorescence spectra of curcumin microcapsules, with the red spectrum referring to the surface region of the capsule (highest fluorescence intensity) and the blue spectrum (lowest fluorescence intensity) referring to the central region of the capsule. (**B**) Confocal microscopy image of 100 µm microencapsulated curcumin.

Figure 6 shows confocal fluorescence microscopy images of microencapsulated curcumin, obtained by the spray-drying process. Figure 6A shows the fluorescence spectra of microencapsulated curcumin with characteristic emission bands between the 540–650 nm regions, obtained through confocal microscopy [41,42].

In Figure 6B, the image obtained by confocal microscopy showed the incorporation of curcumin, with a higher fluorescence intensity on the surface of the microcapsules. The centers of the microcapsules showed low fluorescence intensity, indicating a greater contribution of free curcumin in the surface layer of the capsule. This result can be correlated with the micrograph images obtained by MEV, (Figure 5B), which shows that the particles with an interior may be hollow. The arrangement of the curcumin incorporated into the biopolymer microcapsule may favor the behavior during the release process. Nata and collaborators also observed through imaging a strong fluorescence signal in curcumin microencapsulated by the acetylated starch precipitation method [43].

### 4.5. Thermogravimetric Analysis (TG-DTG)

**Figure 7:**
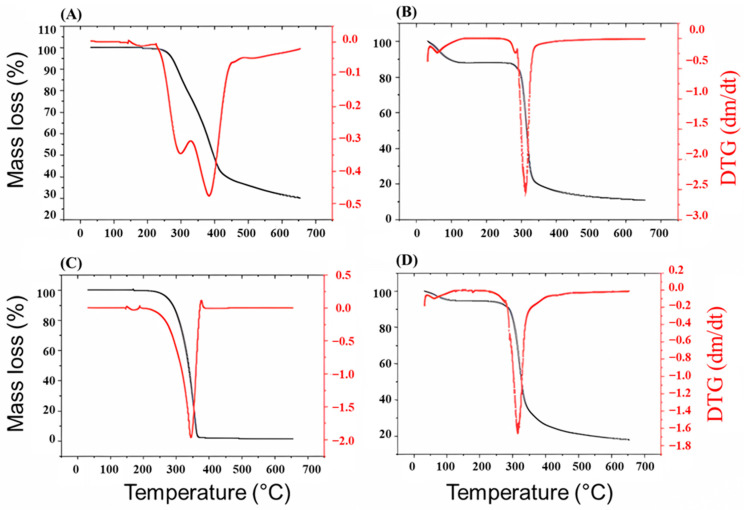
DTG curves (dm/dt) for the materials used in the manufacture of curcumin microcapsules, being the curve (**A**) curcumin, (**B**) soluble starch (**C**) D-mannitol, and (**D**) microencapsulated curcumin.

Figure 7A–D shows the thermogravimetric curves of TG and DTG for free curcumin, starch, D-mannitol and curcumin microcapsules, respectively. The thermogravimetric analyses showed that free curcumin (Figure 7A) presented two mass loss events (%), one of them between 223 and 315 °C (approximately 20%), possibly caused by the decomposition of the substituents of the curcumin molecule, with an average peak at 297 °C in the DTG curve, and the second referring to the complete decomposition of the sample (benzene rings of curcumin) between 315 °C and 422 °C (approximately 38%), with an average peak at 381 °C. The mass loss curve for free curcumin presented a typical behavior for this molecule [44].

Starch (Figure 7B) showed two decomposition events, one of which was related to water loss, between 52 and 126 °C (approximately 8.6%) and the second related to polymer composition between 264 and 347 °C (approximately 67.1%). This event is indicated by the DTG curve by the average peaks at 61 °C and 312 °C. According to the botanical origin of starch and the proportions of amylose and amylopectin, its thermal decomposition can vary between 160 °C and 380 °C [45].

The TG-DTG curves for the biopolymer D-mannitol (Figure 7C), showed that the TG curve shows mass loss that begins at approximately 240 °C, reaching the maximum rate of mass loss at approximately 370 °C. The DTG curve shows an event in the region of 347 °C. These thermal mass loss events are characteristic of the pure D-mannitol molecule [46].

For microencapsulated curcumin (Figure 7D), two events were observed in the TG mass loss curve: the first occurred between 51 and 104 °C (approximately 3%) corresponding to water loss, and the second between 254 and 385 °C (approximately 68.2% mass loss), with a corresponding average peak of 317 °C in the DTG curve (Figure 7D), referring to the decomposition of curcumin. These results showed that both free and microencapsulated curcumin presented high stability against heating. Similar decomposition temperatures for microencapsulated curcumin in excipients such as chitosan [47] and acetylated starch [43] were obtained through different encapsulation methods.

**Figure 8:**
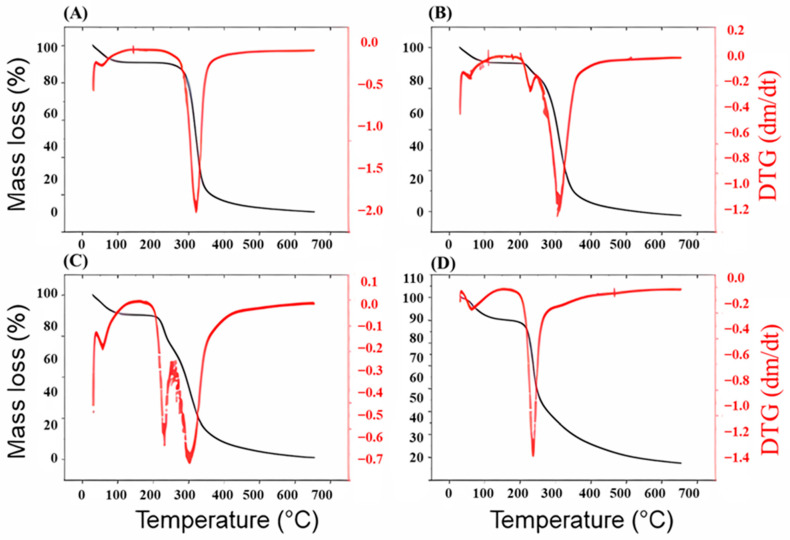
DTG curves (dm/dt) for the materials used in the manufacture of curcumin microcapsules, the curve being (**A**) FCT1, (**B**) FCT2, (**C**) FCT3 e, (**D**) pectin.

The formulations FCT1, FCT2, and FCT3 prepared from microencapsulated curcumin with the addition of different proportions (Table 1) of pectin and starch in their composition were analyzed and the thermogravimetric stability is shown in Figure 8. The thermogravimetric analyses showed that FCT1, which had 60% starch in its composition, presented beginning indicate stability, followed by two mass loss events (%), a phase referring to water loss between 32 and 198 °C (approximately 15.9%), and a second phase, referring to sample decomposition, between 230 and 367 °C (approximately 73.2%) (Figure 8A). The DTG curve for FCT1 presented a more pronounced thermal event, where the greatest mass loss occurred at the temperature of 320 °C.

On the other hand, FCT2 and FCT3 presented three mass loss events (%), the second and third being very similar to those found for free curcumin. Figure 8B shows the thermogravimetric curve for the FCT2 formulation with the addition of 35% pectin and 25% starch in its composition. The curve is similar to FCT1, but variations in mass loss can be noted. There is an event referring to water loss between the regions of 33 and 109 °C, a second event referring to the beginning of sample decomposition at 186–237 °C (3%), and a third thermal event referring to the complete decomposition of the sample between the regions of 237–380 °C, with approximately 64.1% mass loss. This formulation obtained two average temperature peaks in the DTG curve, the first at 231 °C and the second event at 311 °C, respectively.

In Figure 8C, for the FCT3 formulation, the addition of 17.5% pectin and 42.5% starch in its composition presented a thermogravimetric curve similar to FCT1 and FCT2. The loss was that the first thermal event of water loss occurred between the regions of 32–112 °C (8%), and the second event of mass loss (%) in the region of 179 and 241 °C (approximately 10.7%) and the third event referring to the complete loss of mass of the sample between 241 and 402 °C (approximately 48.7%). This higher heating rate may indicate a greater number of compounds that lose mass slowly, such as water, or a change in thermal instability due to the new proportions of the formulation components. For FCT3, two thermal events were obtained with an average between 231 and 305 °C in the DTG curve, respectively.

The pectin showed initial decomposition at 52 to 126 °C with approximately 8% mass loss, probably due to the loss of the adsorbed water molecule, and a second event in the regions between 200 and 250 °C, which can be attributed to the process of depolymerization and decomposition of the polymer [48]. The DTG curve for pectin (Figure 8D) shows a maximum average event in the region of 238 °C [49].

The changes in physical properties may indicate that there was interaction between the excipient starch, D-mannitol, pectin, and the active ingredient microencapsulated curcumin. In all analyses, the initial loss of mass was noted at temperatures below 150 °C, which can be attributed to water evaporation. The curves show that the compositions present characteristic and differentiated thermal behaviors according to the proportions of the excipients in the formulations with modified peaks, suggesting a possible change.

### 4.6. Differential Explanatory Calorimetry (DSC)

**Figure 9:**
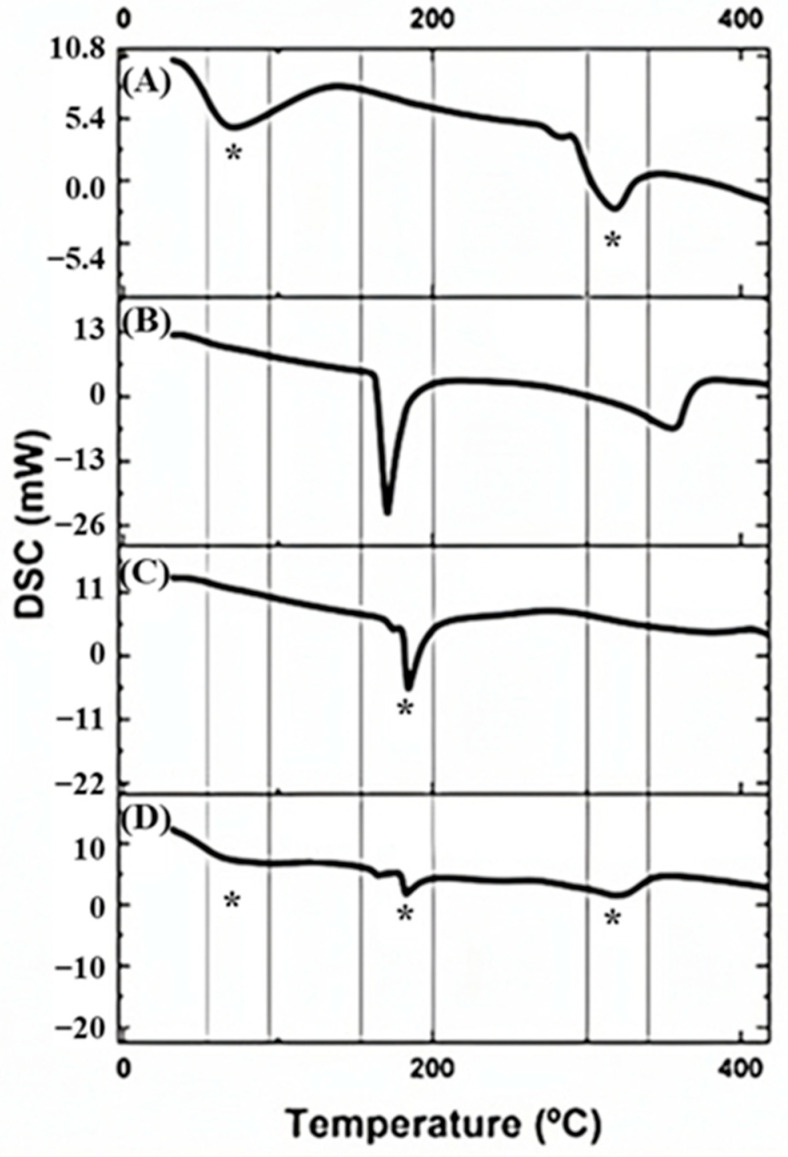
DSC curves: (**A**) starch; (**B**) D-mannitol; (**C**) free curcumin; (**D**) curcumin microcapsule. (*) indicates the common thermal events that occurred for the samples, indicating the compatibility of the components.

Figure 9A–D presents the results obtained by differential scanning calorimetry (DSC) analyses of free curcumin, soluble starch, D-mannitol, and microencapsulated curcumin. The thermogram for soluble starch is presented in Figure 9A, with an endothermic peak in the region of 74 °C, corresponding to the starch gelatinization process [50], and another endothermic peak at 321 °C corresponding to the decomposition of starch [51]. The DSC curve for the biopolymer D-mannitol (Figure 9B) shows a characteristic sharp endothermic peak at 170 °C, indicating the melting behavior of the polymer [52].

The thermogram of free curcumin (Figure 9C) shows a descending (endothermic) peak at 186.4 °C, indicating the melting point and crystalline characteristic of the curcumin molecule [53].

Figure 9D shows the thermogram of microencapsulated curcumin. It is possible to observe that in relation to free curcumin there was a small shift in the endothermic peaks, the first from 186.4 °C to 181 °C. It is noted that the characteristic peak of free curcumin at 186.4 °C had its intensity reduced in the microparticle, which may refer to the curcumin content in relation to the total mass of the sample. The shift in this peak may indicate interactions between curcumin and the excipients. The two thermal events characteristics of the starch polymer were observed in the thermogram for microencapsulated curcumin, demonstrating that curcumin is compatible with this component of the formulation.

**Figure 10:**
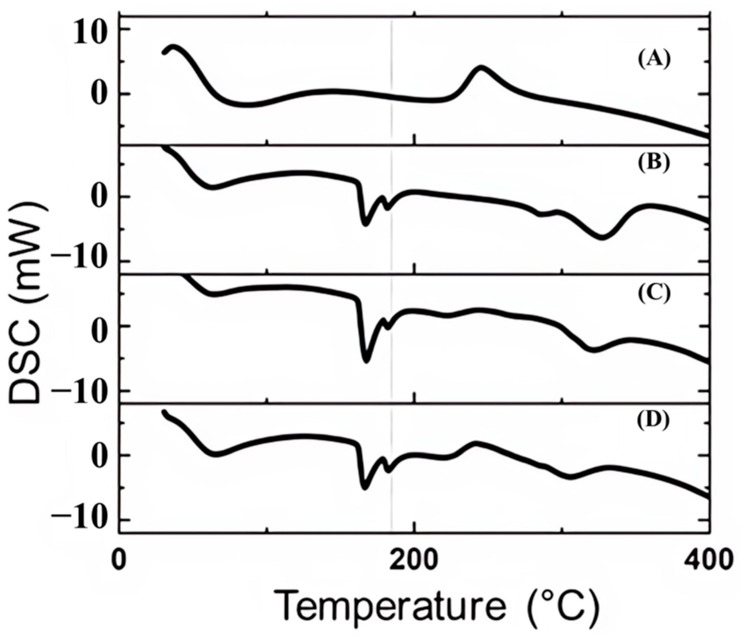
DSC curves for pectin and curcumin formulations: (**A**) pectin; (**B**) FCT1: curcumin, starch, D-mannitol; (**C**) FCT2: curcumin, D-mannitol, pectin, and starch and (**D**) FCT3: curcumin, D-mannitol, pectin, and starch. Dotted line indicates the endothermic peak at 185 °C characteristic of curcumin for FCT1, FCT2, and FCT3, showing possible interaction between excipients and actives.

The DSC analyses for the microencapsulated curcumin formulations FCT1, FCT2, and FCT3 are presented in the thermograms of Figure 10. Figure 10A shows the DSC thermogram curve of the degradation of citrus pectin with an endothermic peak between the heating regions of 50 and 150 °C, characteristic of this type of polysaccharide [54,55]. A second exothermic event indicates the degradation of citrus pectin between the heating regions of 220 and 275 °C [56,57].

In the DSC thermogram for FCT1 (Figure 10B), we can observe the presence of five endothermic events. The first refers to the loss of the water molecules from the sample at 60 °C, from the constituents of the formulation, which may be starch. The second falls in the temperature region of 167 °C, referring to the pattern found for D-mannitol. The third event was at 181 °C, referring to the curcumin pattern. However, it is noted that this event was displaced to a lower temperature, since in the free curcumin curve, the same event was at 186.4 °C. The fourth and fifth events refer to the beginning and complete decomposition of the sample between 287 °C and 326 °C, respectively.

In the DSC thermograms (Figure 10C,D) for the FCT2 and FCT3 formulations, the degradation process was like the FCT1 formulation. It was an exothermic event, characteristic of the pectin polysaccharide at 244 °C. In all formulations, curcumin showed a shift to lower temperatures in relation to the characteristic endothermic event of enthalpy for this free molecule used in this study. Similar behavior was reported for curcumin incorporated in Polyvinylpyrrolidone (PVP k30), which correlates with the increase in solubility during the heating rate [57]. In the case of this study, D-mannitol may have contributed to this effect due to its solubilizing property [58]. This displacement may also indicate that the shape of the crystals is less arranged than in free curcumin or due to the interaction with the polymeric material present in the formulation [59].

The onset of total degradation for FCT2 and FCT3 formulations in the DSC thermogram occurs between temperatures of 306 and 321 °C, respectively. The results of the DSC analyses indicate that it was possible to incorporate curcumin into the components of the formulations, showing affinity between them. These results showed that curcumin is compatible with the components of the formulations, since its characteristics were maintained. The presence and change of thermal events associated with curcumin may also reflect the stabilization of its enol or diketo forms, especially in the encapsulated state, since these tautomers exhibit distinct thermal and spectroscopic signatures [60].

This compatibility refers not only to the preservation of its chemical integrity during processing (MEV results), but also to the maintenance of its tautomeric equilibrium (keto–enol forms), which is critical for the stability and bioactivity of curcumin [25,61]. Spectroscopic and thermal analyses confirmed that no significant changes occurred, suggesting that the microenvironment created by the encapsulating agents protects the active form of curcumin.

### 4.7. Infrared Spectra (FTIR)

**Figure 11:**
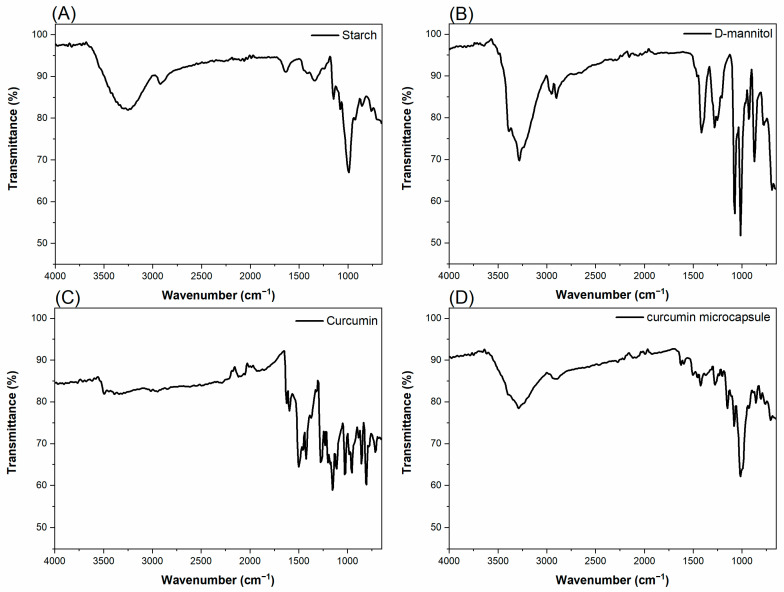
FTIR spectra of the encapsulant, active and microcapsule materials: (**A**) starch, (**B**) D-mannitol, (**C**) curcumin and (**D**) microencapsulated curcumin.

Figure 11 shows the infrared spectra (FTIR) for (A) starch, (B) D-mannitol, (C) curcumin, and (D) curcumin microcapsule, respectively.

Figure 11A shows the FTIR spectrum of starch, with characteristic bands in different regions. A broad band is observed between 3630–3000 cm^−1^, attributed to the stretching vibration of the O-H bond. A peak at 2930 cm^−1^ corresponds to the asymmetric stretching of C-H bonds. In the region between 1700–1560 cm^−1^, there are signals related to the CH_2_ bending mode. The band at 1411 cm^−1^ can be associated with the CH_2_ scissor mode and the C-H and C-O-H deformations. At 1344 cm^−1^, a band corresponding to the bending of the C-O-H group and the torsion of CH_2_ is observed. The signal at 1238 cm^−1^ refers to the vibrational modes related to CH_2_. The 1146 cm^−1^ region indicates the presence of vibrations associated with C-O stretching and C-O-H bending. An intense peak between the 1077 and 994 cm^−1^ regions is related to C-O-C vibrations, typical of the glycosidic ring structure. The bands at 921 cm^−1^ and 860 cm^−1^ are attributed, respectively, to deformations of the C-H/CH_2_ bonds and to the stretching of the C-C bond, characterizing the structure of the polysaccharide [62,63].

The FTIR spectrum of D-mannitol (Figure 11B) shows that characteristic bands in the regions of 3385, 3280 and 3250 cm^−1^ are attributed to the stretching vibrations of the O-H bonds. The peaks at 2989, 2950, and 2900 cm^−1^ correspond to the stretching vibrations of the C–H bonds. The band at 1411 cm^−1^ refers to the scissor-type deformation mode of the CH_2_ group, while the peak at 1283 cm^−1^ is related to the in-plane deformation of the O-H groups. In the region between 1087 and 1013 cm^−1^ the peaks are attributed to the stretching of the C-O bonds. The peaks in the regions of 960, 926, 875, and 777 cm^−1^ correspond to weak vibrations associated with deformations outside the C-C plane [19,64,65,66].

The FTIR spectrum of curcumin (Figure 11C) presents characteristic bands with bands at 3495 and 3385 cm^−1^ attributed to the stretching of the O-H bonds of the phenolic hydroxyls. The peak at 1628 cm^−1^ corresponds to the stretching of the C=O bond of the aromatic portion of the ketone β-diketone. The peak at 1597 cm^−1^ is related to the C=C stretching vibrations of the benzene ring. The band at 1509 cm^−1^ refers to the stretching of the C=O bond of the conjugated enone system and vibrations associated with C=C. The bands at 1460 and 1420 cm^−1^ are attributed to the angular deformation vibrations of the C-H bonds. The region between 1270–1200 cm^−1^ presents stretching bands of the C-O bonds of the phenolic groups. Between 1156–1110 cm^−1^, bands are attributed to the stretching of C-O and C-O-C bonds, present in ethers and phenols. In the region between 954–807 cm^−1^, the peaks are related to the out-of-plane bending modes of the CH group of the aromatic ring [19,67,68,69].

The FTIR spectrum of microencapsulated curcumin (Figure 11D) shows the main vibrational peaks of curcumin, but with lower intensity, which suggests a possible interaction between the active molecule and the excipients of the polymer matrix. The bands associated with O-H stretching, originally observed at 3495 and 3385 cm^−1^, appear shifted to 3397 and 3269 cm^−1^, indicating possible formation of hydrogen bonds with starch and/or D-mannitol. The peaks without 1625 and 1597 cm^−1^, attributed to the carbonyl group of the β-diketone moiety and the aromatic ring of curcumin, were maintained, but with slight shift and attenuation. Furthermore, characteristic bands of the excipients in the microcapsule are observed, such as the band at 1353 cm^−1^, attributed to D-mannitol, and intense peaks in the region of 1138 to 1017 cm^−1^, with overlapping C-O vibrations of alcohols (present in starch and D-mannitol). Such changes in the spectrum indicate evidence of curcumin encapsulation and suggest physical or chemical interactions between the components of the formulation.

**Figure 12:**
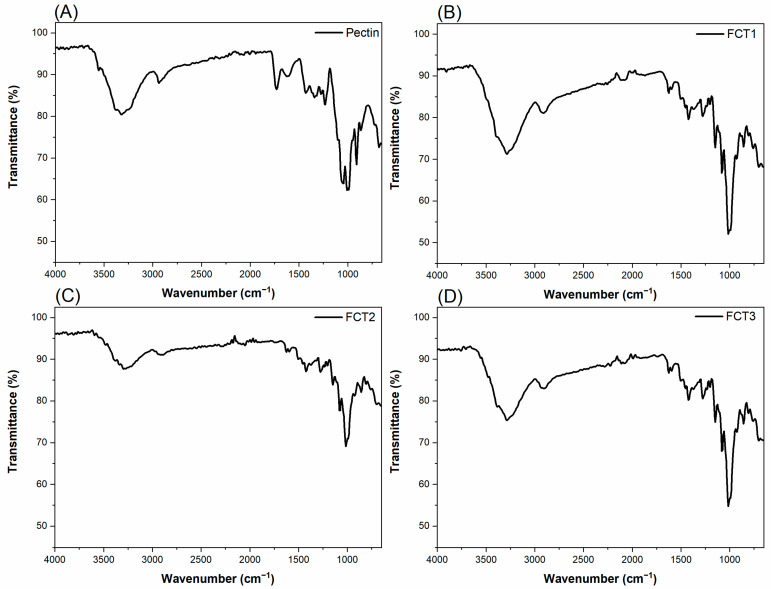
FTIR spectra of pectin and microencapsulated curcumin formulations: (**A**) pectin; (**B**) FCT1: curcumin, starch, D-mannitol; (**C**) FCT2: curcumin, D-mannitol, pectin, and starch and (**D**) FCT3: curcumin, D-mannitol, pectin, and starch.

Figure 12 shows the FTIR spectra for the excipient pectin and the formulations with microencapsulated curcumin tablets: (A) pectin; (B) FCT1: curcumin, starch, D-mannitol; (C) FCT2: curcumin, D-mannitol, pectin and starch, and (D) FCT3: curcumin, D-mannitol, pectin and starch, respectively.

The FTIR spectrum of pectin (Figure 12A) presents characteristic bands: a broad band between 3528–3000 cm^−1^ is attributed to the stretching of O-H bonds. The peaks between 2989–2923 cm^−1^ refer to the stretching vibrations of C-H bonds. The absorption at 1633 cm^−1^ corresponds to the asymmetric stretching of the C=O bond of the carboxylate groups. The peaks in the range of 1441–1338 cm^−1^ are associated with the angular deformation of the C-H bonds and the symmetric vibration of the C=O bond of the carboxylic groups. In the region between 1233–1149 cm^−1^, the peaks are attributed to the stretching of the C-O-C and C-C bonds, corresponding to the structure of the glycosidic ring. The peak at 1078 cm^−1^ is related to the stretching of C-O bonds, while the bands between 994–921 cm^−1^ are attributed to the deformations of C-H bonds, characteristic of the α and β configurations of pectin [70,71].

The comparative analysis of the FTIR spectra of the FCT1, FCT2, and FCT3 formulations demonstrated that, although there are no significant shifts in the band positions, there is a variation in the relative intensity of the peaks, directly related to the proportion of excipients, especially starch and pectin. The FCT1 formulation, with a higher starch content (60%), presented more intense bands at 1078 and 990 cm^−1^, attributed to C-O-C stretching vibrations, typical of the glycosidic ring structure. Other notable bands include 1011, 1150, and 1625 cm^−1^, all associated with the polysaccharide structure of starch. In the FCT2 formulation, with a lower starch concentration (25%) and a higher proportion of pectin, a reduction in the intensity of the bands attributed to starch was observed, accompanied by an increase in intensity in the regions of 3555 cm^−1^ (O-H elongation), 1630 cm^−1^ (asymmetric C=O elongation of carboxylates), and 1050 cm^−1^ (C-O and C-O-C), characteristic of pectin. FCT3, with an intermediate composition (42.5% starch), exhibits spectral intensities corresponding to the equilibrium between the two excipients. Furthermore, in all formulations, bands characteristic of curcumin were observed, such as 1625, 1590–1597, and 1503–1509 cm^−1^, indicating the presence of the active molecule and suggesting that its chemical structure was preserved after the encapsulation process. The observations indicate that, although the chemical composition of the formulations remains similar, variations in the proportions of starch and pectin directly affect the intensity of the FTIR bands, without indicating new chemical interactions, but reinforcing the successful incorporation of the excipients into the microcapsule matrix.

The integrated analysis of morphological, thermal, and spectroscopic techniques allowed a comprehensive understanding of the effectiveness of the curcumin microencapsulation process. SEM images showed the formation of predominantly spherical particles with evidence of a hollow interior, while confocal microscopy confirmed the presence of curcumin, mostly distributed on the surface of the microcapsules. Thermal analyses (TG/DTG and DSC) demonstrated that the encapsulated curcumin presented greater thermal stability in relation to the free form, with shifts in the degradation and fusion events, indicating physical interaction with the excipients and modification of the crystalline state. The formulations with higher starch content (FCT1) presented greater thermal resistance, while those with more pectin (FCT2) showed additional events, compatible with the contribution of this excipient. The FTIR data reinforced these findings by revealing the preservation of the characteristic bands of curcumin, with attenuation of intensity and discrete shifts, suggesting interactions via hydrogen bonds with matrix components. Overall, the results confirm that microencapsulation was successful, promoting thermal stabilization of curcumin, its structural protection and compatibility with excipients.

### 4.8. Photodynamic Inactivation of Ae. aegypti Larvae

#### 4.8.1. PDI Analysis: Larvicidal Efficacy

The results of larval mortality of the *Ae. aegypti* after 24 h of exposure and the lethal concentration values (LC_50-24h_) are presented in Table 2.

Among the formulations, FCT2 was the most efficient, as it presented the lowest LC_50_. The LC_50-24h_ value for microcapsules was 0.40 mg/L, while for free curcumin it was 23.02 mg/L. These results showed a strong larvicidal potential for curcumin microcapsules, being approximately 57 times more effective than free curcumin. This result may indicate that the curcumin microencapsulation technique has improved its application due to its low solubility and stability. This improvement can be associated with the stabilization of its tautomeric forms, which play a fundamental role in its solubility and photodynamic activity. The encapsulation likely preserved the active isomeric configuration of curcumin, preventing degradation and enhancing its environmental performance [25,61]. In addition, according to Guo [27] it used two encapsulation methods, and both exhibited improved stability against heat and acidity and improved stability.

Our results also showed much lower values than the studies by Mezzacappo and collaborators [72], whose LC_50-24h_ value for third-instar larvae was 60 mg/L. It is also worth mentioning that the microcapsules were more efficient against *Ae. aegypti* than other plant-derived compounds and essential oils, such as *Machaerium acutifolium* stem extract (LC_50_: 205 mg/mL) [73], n-hexane fraction of crude stem extract (S-HEX) of *Ocotea nutans* (LC_50_: 14.14 mg/mL) [74], crude methanol extract of *Calpurnia aurea* (LC_50_ of 84.85 mg/mL) [75].

These results indicate that microencapsulation not only increases the solubility of curcumin but also improves its bioavailability within the larvae, increasing photodynamic efficacy.

#### 4.8.2. PDI Analysis: Laboratory Persistence

**Figure 13:**
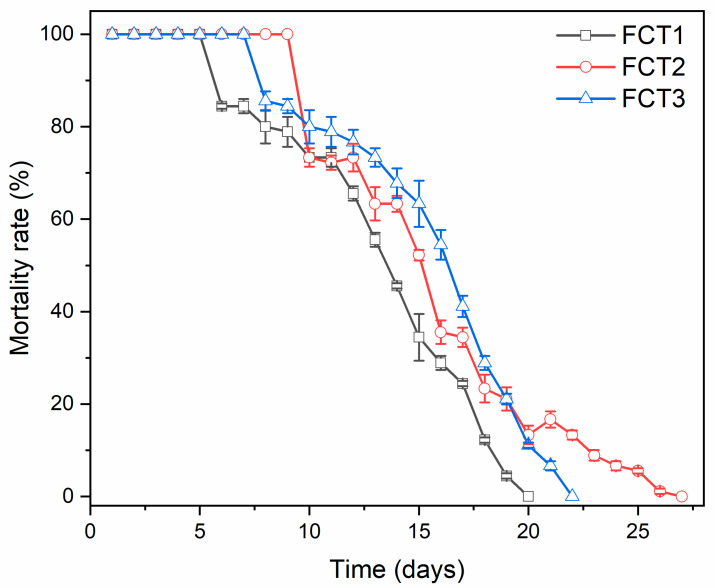
Residual activity of microencapsulated curcumin formulations (FCT1: curcumin, starch, D-mannitol), (FCT2: curcumin, D-mannitol, pectin, and starch) and (FCT3: curcumin, D-mannitol, pectin, and starch) by time in days in *Ae. aegypti* under laboratory conditions.

The laboratory residual activity results of the microencapsulated curcumin formulation are shown in Figure 13. FCT1 had 100% larval mortality in only 5 days and had a residual effect of 20 days. The FCT2 formulation showed 100% larval mortality up to 10 days of exposure to irradiation. Then, there was a 50% decay at 15 days and its residual effect ended at 27 days. And FCT3 had 100% mortality for 7 days and the residual effect ended at 22 days.

Comparing the three curcumin formulations (FCT1, 2 and 3), FCT2 showed a better residual effect because it presented 5 more days of residual effect than FCT1 and 3 more days than FCT3. Compared to the cardanol fraction (derived from cashew nut shells), a very promising molecule for eliminating larvae, the formulation was approximately 7 times more persistent. Cardanol has an average residual activity time of 3.7 days [76].

When analyzing the current literature, there is only one study of the residual activity of curcumin on *Ae. aegypti* larvae, with physical mixtures of curcumin and D-mannitol, showing residual activity of only 5 days [72], indicating that the microencapsulated curcumin formulation presented a longer period with its residual effect enhanced. The microencapsulation of the photosensitizing active ingredient curcumin in carrier agents D-mannitol and starch improved its stability, thus ensuring a longer time of photolarvicidal efficacy.

#### 4.8.3. Confocal Microscopy Larvae *Ae. aegypti* Formulation

**Figure 14:**
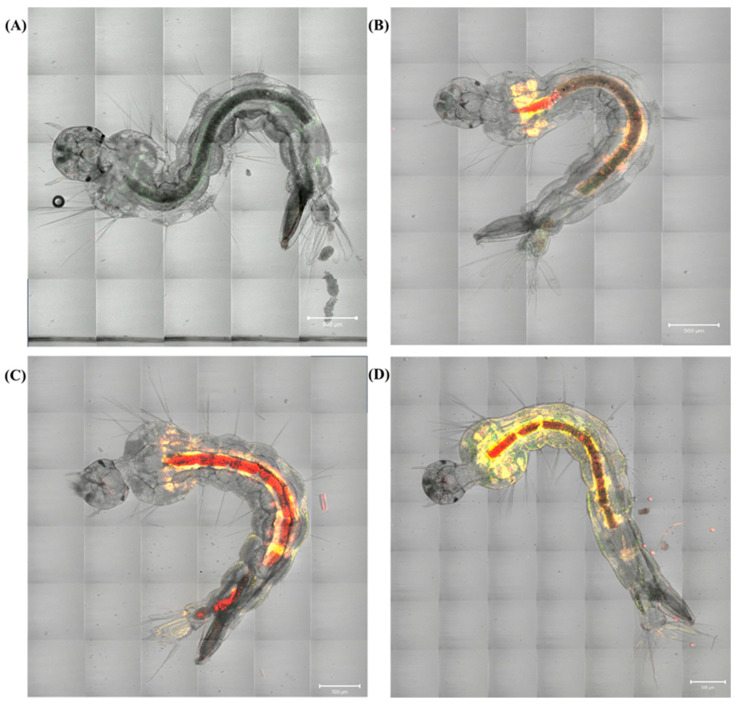
Confocal microscopy images of *Ae. aegypti* larvae with 20 min of exposure to formulations: (**A**) control (**B**) FCT1 (curcumin, starch, D-mannitol), (**C**) FCT2 (curcumin, D-mannitol, pectin, and starch) and (**D**) FCT3 (curcumin, D-mannitol, pectin, and starch). Scale bar = 500 μm.

Confocal microscopy was used to evaluate the penetration and distribution of curcumin formulations in *Ae. aegypti* larvae, aiming to understand the differences in LC_50_ observed in the PDI process: FCT1, FCT2, and FCT3 values (Table 2). In laboratory persistence tests, the FCT2 formulation demonstrated greater duration over the days, standing out in relation to the others. In addition, FCT2 presented a significantly lower LC_50_ when compared to FCT1 and FCT3, indicating greater efficiency. Figure 14 shows tile scan images of larvae in the 3rd developmental stage of *Ae. aegypti* (A) control, (B) FCT1, (C) FCT2, and (D) FCT3.

Figure 14A shows control *Ae. aegypti* larvae, not exposed to curcumin formulations. Figure 14B–D show *Ae. aegypti* larvae after 20 min of exposure to the different curcumin formulations with larval tissues. The technique allows the visualization of internal and external structures of the larvae in high resolution. The distribution of curcumin in the exposed larvae is uniform throughout the thoracic region and abdominal segments. This pattern of rapid incorporation and wide distribution of curcumin in the *Ae. aegypti* larvae is consistent with data in the literature [15,77,78,79].

In Figure 14B–D shows confocal microscopy images of *Ae. aegypti* larvae exposed for 20 min to the formulations FCT1, FCT2, and FCT3, and the emission spectral profiles of curcumin for each of the formulations, respectively. The bioavailability of curcumin in the digestive systems of *Ae. aegypti* larvae may contribute to explaining the form of absorption and metabolism, where fluorescence indicates the presence of the compound in specific locations. Figure 14B shows an image of *Ae. aegypti* larvae, where it is possible to observe the incorporation of FCT1, starting in the thorax region and continuing to the abdominal segment.

**Figure 15:**
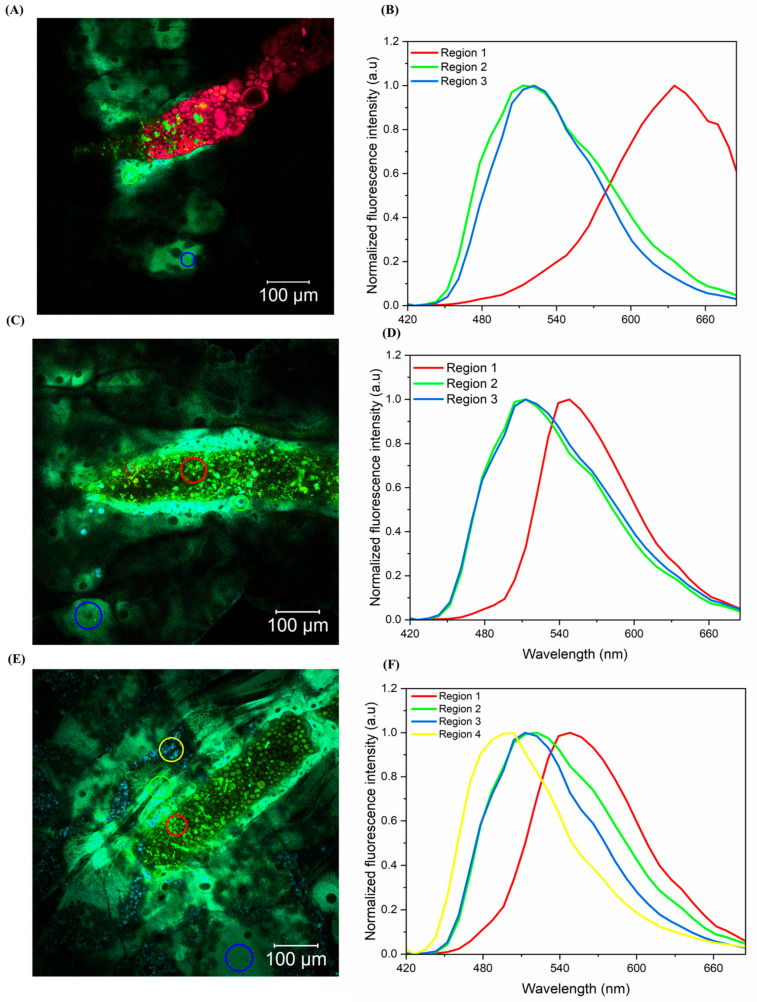
Confocal microscopy images of *Ae. aegypti* larvae with 20 min of exposure to formulations: (**A**) FCT1 (curcumin, starch, D-mannitol), (**B**) emission spectrum of curcumin in FCT1 (**C**) FCT2 (curcumin, D-mannitol, pectin, and starch), (**D**) emission spectrum of curcumin in FCT2, (**E**) FCT3 (curcumin, D-mannitol, pectin, and starch) and (**F**) emission spectrum of curcumin in FCT3.

Figure 15 represents the region of the digestive tract of the *Ae. aegypti* larva exposed for 20 min to the curcumin formulations: FCT1 (12A), FCT2 (12B), and FCT3 (12C) and their emissions spectra in the highlighted regions: FCT1 (12B), FCT2, (12D), and FT3 (12F).

In Figure 15A,B, region 1 indicates greater accumulation of curcumin or a specific interaction with biomolecules in the digestive tract, such as lipids and proteins. Region 2 indicates a more uniform distribution, while region 3 is associated with less intense regions. In region 1, the displacement may be associated with the location, where the pH is more basic because it is the larva’s digestive tract and curcumin can form ionic complexes, significantly altering its optical properties, also showing that curcumin is fully bioavailable [80]. Region 2 is in the basal cells of the intestinal epithelium and region 3 is located in the tissues deeper to the larva tract. Both regions (2 and 3) have a more neutral pH, so the ketone form of curcumin dominates [80]. And the intensity may be related to the distance of curcumin when ingested by the larva interacting with the cells and the ability to permeate the membranes, since region 2 is closer than region 3.

In Figure 15C,D, the fluorescence spectra are more intense and uniform throughout the larval tract (regions 1, 2, and 3), indicating greater distribution and interaction of curcumin with the tissues. This greater bioavailability of curcumin may have led to greater damage to the cells of the larval tract. Therefore, this effect may be related to the results of the PDI assays, in which FCT2 was shown to be more efficient than the others.

In Figure 15E,F, the fluorescence spectra in region 1 continues to show a peak at ~600 nm, indicating a change in the peak due to contact with the basic pH of the larval tract [80,81]. Regions 2 and 3 remain similar to the previous spectra (Figure 15B,D), suggesting that these regions contain curcumin distributed uniformly and that its form does not change chemically. In addition to the three regions, there is a new region (Region 4), indicating an additional fluorescent pattern. The fluorescence is more dispersed, showing changes in the chemical state of curcumin due to the difference in the microenvironment.

### 4.9. Release in Static Condition of Formulated Curcumin

**Figure 16:**
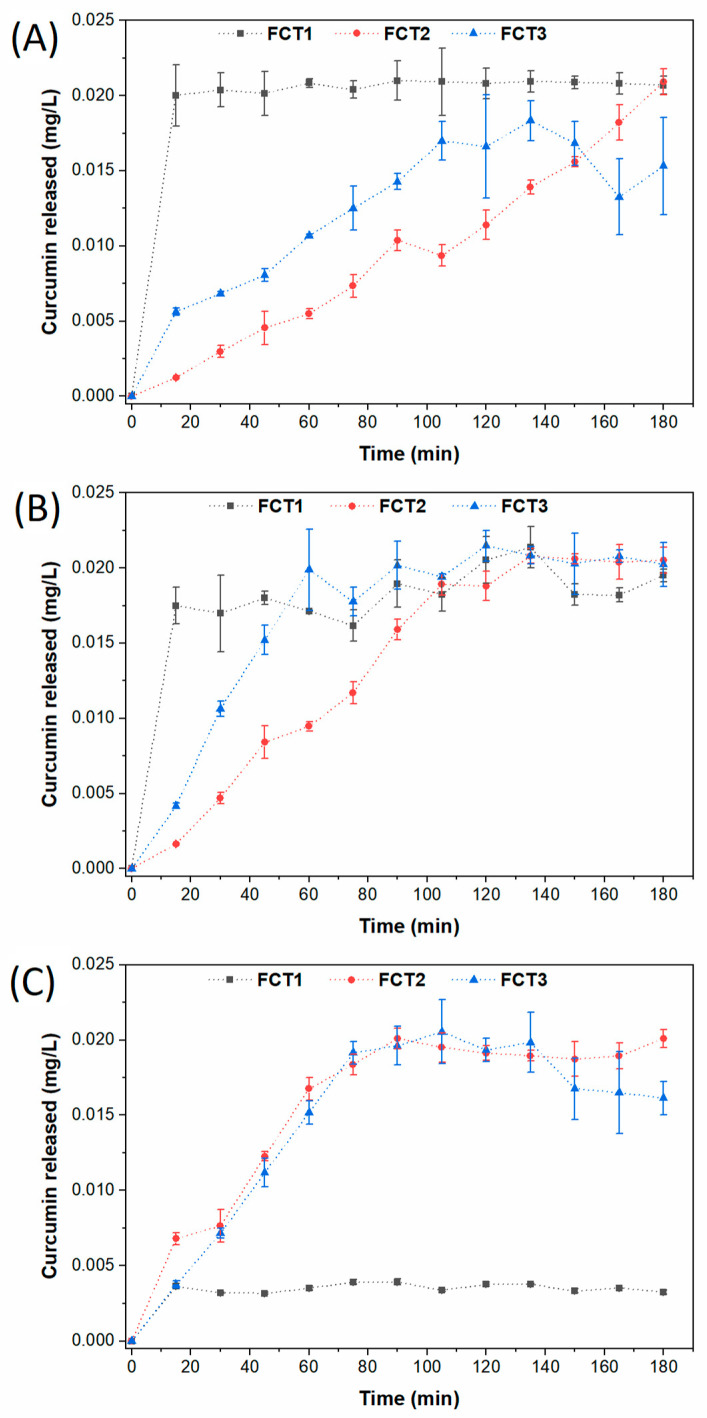
Release curves for microencapsulated curcumin formulations (FCT1: curcumin, starch, D-mannitol), (FCT2: curcumin, D-mannitol, pectin and starch) and (FCT3: curcumin, D-mannitol, pectin and starch) as a function of pH = 3 (**A**), pH = 7 (**B**) and pH = 11 (**C**).

The release kinetics were investigated to understand the release mechanisms of curcumin from microparticles based on FCT1, FCT2, and FCT3 formulation as a function of pH. Figure 16 shows the release profile of curcumin from microparticles up to its maximum released concentration at pHs 3 (Figure 16A), 7 (Figure 16B), and 11 (Figure 16C). In addition to affecting matrix degradation, the pH of the environment can influence the tautomeric equilibrium of curcumin. In acidic conditions, the keto form is often favored, while in neutral to basic environments, the enol form becomes more prevalent. These shifts may alter curcumin’s interaction with both the matrix and the biological system [60].

For the pH 3 (Figure 16A), it is possible to observe that the formulations presented different release kinetics. FCT1 presented a fast release, reaching maximum release in 15 min and then remaining constant. FCT2 presented a slower release profile than the others and FCT3 presented a moderate release profile between FCT 1 and 2.

It is possible that FCT1 presents a rapid release in an acidic medium (pH 3) because it has the highest percentage of starch (60%). The starch’s ability to form gels or thicken solutions may be reduced, as the acid weakens the bonds between the amylose and amylopectin chains, making it difficult to form a robust structural network and faster degradation [54]. This degradation can generate a faster release of curcumin into the environment (Figure 16A).

At pH 7, FCT1, due to the lack of pectin, did not control the release (Figure 16B), since pectin is an excipient that aims to control the drug release profile, especially in controlled release systems. And at pH 11, there was rapid degradation of curcumin, since in an alkaline medium there is deprotonation and rapid formation of degradation products such as feruloylmethane and ferulic acid [82].

FCT2 presented the most controlled release, as it has the highest percentage of pectin (30%). Pectin has a polysaccharide structure and the ability to form gels in an aqueous medium, and is widely used as support or wall materials, in addition to being able to be used to delay release [54,83]. In addition, it can be sensitive to pH. In the acidic environment, it can protect the drug, curcumin reached the highest concentration only after 180 min (Figure 16A), while in neutral or basic pH, it can disintegrate or be degraded, promoting the release of the active a little faster, 135 min for pH 7 (Figure 16B) and 90 min for pH 11 (Figure 16C).

Finally, FCT3 presented a more controlled release than FCT1 because it has a percentage of pectin in its formulation (17.5), but it is lower than FCT2. This factor may be related to the properties of pectin [54,83]. Furthermore, unlike FCT2, FCT3 at pH 11 presented manipulation after 150 min (Figure 16C).

The release profiles of FCT2 were analyzed by applying the Korsmeyer–Peppas [84] mathematical model using a simple empirical model [*f* = *kt^n^*] [84,85,86] to calculate the release constants (*k*) and the correlation coefficients (R^2^). Results are summarized in Table 3. The mathematical model presented a good adjustment to the experimental curves resulting in R^2^ from 0.96 to 0.98. The kinetic constant *k* is characteristic for a particular system considering structural and geometrical aspects; *n* is the release exponent representing four different mechanisms (Fickian diffusion, anomalous transport, Case-II transport, and Super Case-II transport) [87,88] considering spherical particles, and *t* is the release time.

The releasing mechanism by Fickian diffusion is the mechanism in which the active diffusion through the particle is exclusively determined by Fickian diffusion. In the case of anomalous transport, the active release is due both to Fickian diffusion and swelling/relaxation of the carrier. The Case-II transport is controlled by the swelling and relaxation of carriers and it is independent of time. In the Super Case-II transport, the release is ruled by the macromolecular relaxation of the polymeric chains [84].

In general, the *n* value determines the dominant release mechanism. Considering spherical particles, *n* = 0.43 represents a Fickian diffusion; 0.43 ≤ *n* ≤ 0.85 represents an anomalous transport. When *n* = 0.85, the release is ruled by the Case-II transport, and *n* > 0.85 is related to a Super Case-II transport [89]. Our results showed that curcumin was released from microparticles by different mechanisms as a function of pH medium: at pH = 3 and pH = 11, curcumin was released by the Super Case-II transport mechanism (*n* = 1.19 and 1.0, respectively), while at pH = 7 (*n* = 0.79) the anomalous transport mechanism was observed.

**Figure 17:**
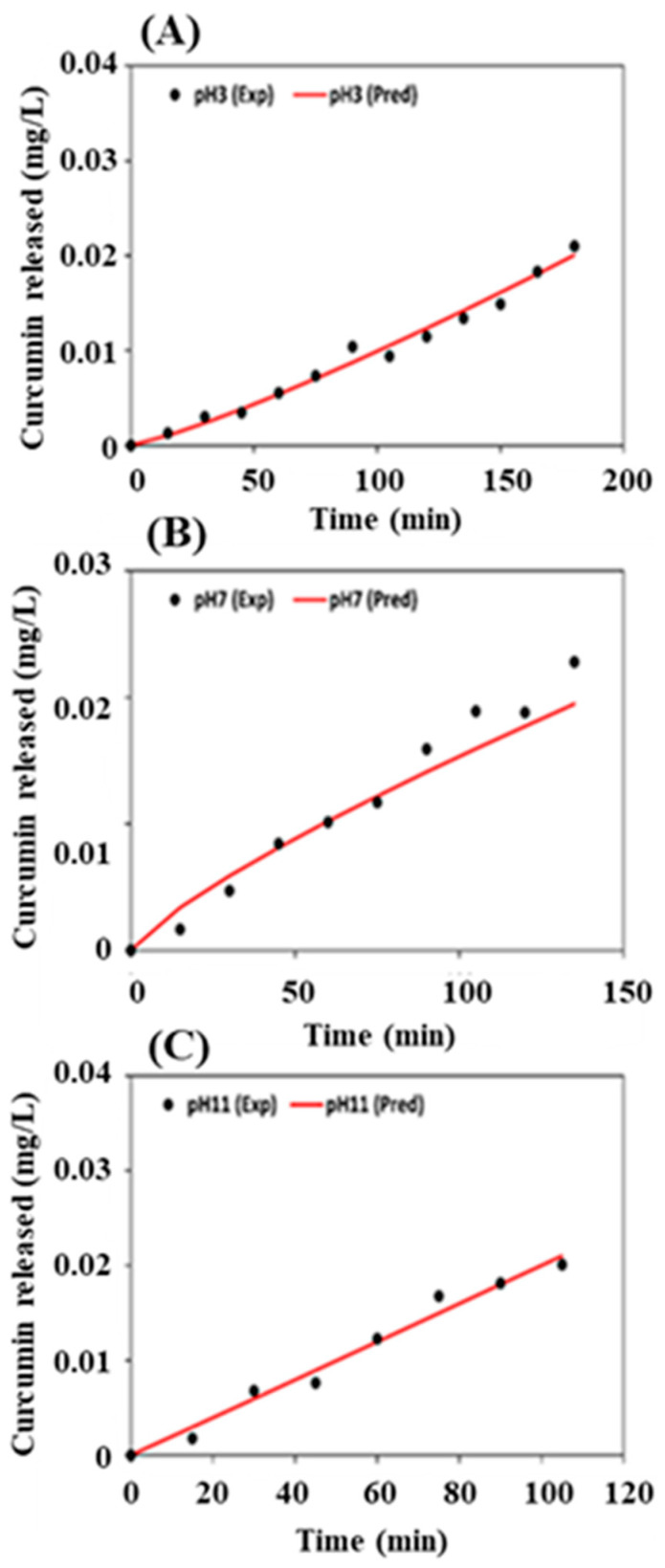
Controlled release curves adjusted to the Korsmeyer–Peppas model [released curcumin (mg L^−1^) *versus* time (min)] for the FCT2 formulation as a function of (**A**) pH = 3, (**B**) pH = 7 and (**C**) pH = 11.

The release profiles revealed a dependence between the maximum released concentration of curcumin and the maximum release time the Figure 17. The FCT2 system released a similar concentration of curcumin (0.02 mg/L) in the three evaluated pH medium. However, the time required for this released concentration to be reached was different: the release time increased when the pH of the medium decreased from 11 to 3.

Considering the release at pH = 11, the concentration of 0.02 mg/L was reached after 105 min. Similar concentration of curcumin was released after 135 min at pH = 7, and after 180 min at pH = 3. The difference of the maximum time releasing may be associated to the solubility of the microparticles carrier at different pH medium. From pH = 3 to pH = 11, a decrease of 75 min was observed for the total release (0.02 mg/L) of curcumin.

Considering that the microparticles carriers of the FCT2 formulation are constituted of D-mannitol (30%), starch (25%) and pectin (35%), the better solubility of D-mannitol at basic pH may have influenced the release of curcumin in shorter releasing time. The aqueous solubility of a 4-methoxybenzeneboronic acid as a function of pH both in the absence and in the presence of varying D-mannitol concentration was evaluated by Lopalco and our collaborators [89]. The authors observed that the concentration of D-mannitol influenced the solubility of the acid from neutral to acid pH values. However, from neutral to basic pH, the solubility was considerably increased. For this reason, the FCT2 formulation can release curcumin in shorter times at pH = 11 according to the Super Case-II release mechanism.

### 4.10. Photostability Analysis of Microencapsulated Curcumin Formulations

**Figure 18:**
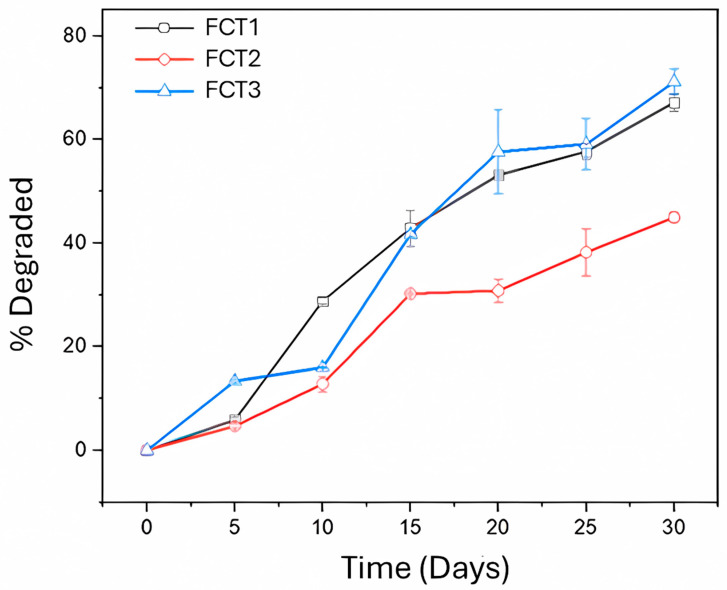
Photodegradation of microencapsulated curcumin formulations (FCT1: curcumin, starch, D-mannitol), (FCT2: curcumin, D-mannitol, pectin, and starch) and (FCT3: curcumin, D-mannitol, pectin, and starch) exposed to white, fluorescent lights.

Figure 18 shows the percentage degradation of the active ingredient curcumin in the formulations as a function of irradiation and over 30 days.

For FCT1, the degradation of the active ingredient is increasing, reaching a percentage of 67% over 30 days. However, for FCT2, the photodegradation process of the active ingredient curcumin occurs more slowly, with approximately 47% after 30 days of exposure to light. The same behavior was observed in FCT1 for FCT3, corroborating the release results, in which FCT2 presented a more controlled release than the others.

Furthermore, the FCTs of the present study were more stable than other curcumin formulations combined with D-mannitol. Garbuio and collaborators [19] presented a curcumin formulation with D-mannitol (50:50 *w*/*w*) that after 24 h showed a photodegradation of just over 49% when exposed to white, fluorescent light (18 W, intensity of 0.83 mW/cm^2^). Mezzacappo and collaborators [76] showed that in a formulation of 99% D-mannitol and 1% curcumin, the photodegradation under white, fluorescent light (18 W, 0.5 mW/cm^2^) after 48 h was 67% and reached 100% after 14 days.

## 5. Conclusions

This study demonstrated the potential of microencapsulated curcumin as an effective photoinsecticidal agent for *Ae. aegypti* control. The formulation obtained by spray drying, using D-mannitol and starch as encapsulating agents, was later modified with the addition of pectin. The results showed that this modification significantly improved the solubility, stability, and controlled release profile of curcumin. Among the evaluated formulations, FCT2 exhibited the highest larvicidal efficacy, with a 57-fold reduction in LC_50_ compared to free curcumin and maintained its residual effect for up to 27 days, highlighting its potential for prolonged vector control.

The pH-dependent release profile confirmed that the formulation could modulate curcumin availability under different environmental conditions, which is crucial for optimizing its efficacy in breeding sites. Confocal microscopy analysis further revealed the efficient absorption and bioavailability of curcumin in larval tissues, reinforcing its mechanism of action in photodynamic inactivation. These findings suggest that curcumin-based photoinsecticidal formulations could serve as a sustainable and environmentally friendly alternative to conventional insecticides, mitigating issues related to chemical resistance and environmental toxicity. Future studies should explore large-scale applications, field trials, and further optimization of formulations to enhance stability and economic feasibility under real conditions. The results also emphasize the importance of preserving curcumin integrity during formulation, as this structural feature underlies its physicochemical properties and photodynamic performance.

## Figures and Tables

**Table 1:** Formulation proportions obtained by physical mixing of spray-dried microencapsulated curcumin powder with the excipients D-mannitol, pectin, and starch.

	Curcumin (%)	D-Mannitol (%)	Pectin (%)	Starch (%)
FCT1	10	30	-	60
FCT2	10	30	35	25
FCT3	10	30	17.5	42.5

**Table 2:** Photolarvicidal activity of Curcumin, microcapsule of curcumin, curcumin formulations (FCT1: curcumin, starch, D-mannitol), (FCT2: curcumin, D-mannitol, pectin, and starch) and (FCT3: curcumin, D-mannitol, pectin, and starch). on *Ae. aegypti* larvae after 24 h.

Formulations	LC_50-24h_ (mg/L)	95% Confidence Limit LC_50_ (Lower-Upper)	R^2^
Curcumin	23.02	(17.100–32.180)	0.991
Microcapsule of curcumin	0.40	(0.283–0.604)	0.990
FCT1	1.07	(0.805–1.431)	0.987
FCT2	0.27	(0.068–0.335)	0.998
FCT3	1.89	(0.921–2.452)	0.985

LC_50_, Lethal concentration 50% mortality (in 24 h); confidence interval of 95% (values in parentheses represent lower and upper confidence limits of the interval); R^2^, R-square.

**Table 3:** Coefficients obtained from the controlled release according to the Korsmeyer–Peppas mathematical models.

pH	k (mg/L/min)	*n*	R^2^	T_max_ (min)	C_max_ (mg/L)	Release Mechanism
3	4 × 10^−5^	1.19	0.98	180	0.02	Super Case-II
7	3.9 × 10^−4^	0.79	0.96	135	0.02	Anomalous transport
11	1.93 × 10^−4^	1	0.98	105	0.02	Super Case-II

*k* is characteristic for a particular system considering structural and geometrical aspects; *n* is the release exponent; T_max_ is the time (min) to release the maximum concentration of curcumin and C_max_ is the maximum released curcumin concentration.

## Data Availability

The data are not publicly available due to ethical and confidentiality restrictions.

## Supplementary Materials

The following supporting information can be downloaded at: https://www.mdpi.com/article/10.3390/pharmaceutics17040496/s1, Figure S1: Curve calibration curcumin, concentration 0.0–7.0 m/L and linear correlation R^2^ 0.99993.

## References

[1] Donalisio M.R., Freitas A.R.R., Zuben A.P.B.V. (2017). Arboviruses emerging in Brazil: Challenges for clinic and implications for public health. Rev. Saude Publica.

[2] Patterson J., Sammon M., Garg M. (2016). Dengue, Zika and Chikungunya: Emerging Arboviruses in the New World. West. J. Emerg. Med..

[3] Gan S.J., Leong Y.Q., bin Barhanuddin M.F.H., Wong S.T., Wong S.F., Mak J.W., Ahmad R.B. (2021). Dengue fever and insecticide resistance in aedes mosquitoes in Southeast Asia: A review. Parasit. Vectors.

[4] Santos L.L.M., de Aquino E.C., Fernandes S.M., Ternes Y.M.F., Feres V.C.d.R. (2023). Dengue, Chikungunya, and Zika virus infections in Latin America and the Caribbean: A systematic review. Rev. Panam. De Salud Pública.

[5] Phillips M.L. (2008). Dengue Reborn: Widespread resurgence of a resilient vector. Environ. Health Perspect..

[6] Wilson A.L., Courtenay O., Kelly-Hope L.A., Scott T.W., Takken W., Torr S.J., Lindsay S.W. (2020). The Importance of vector control for the control and elimination of vector-borne diseases. PLoS Negl. Trop. Dis..

[7] Dias Í.K.R., Martins R.M.G., Sobreira C.L.d.S., Rocha R.M.G.S., Lopes M.d.S.V. (2022). Ações educativas de enfrentamento ao *Aedes aegypti*: Revisão integrativa. Cien Saude Colet..

[8] Mahmoudi H., Bahador A., Pourhajibagher M., Alikhani M.Y. (2018). Antimicrobial photodynamic therapy: An effective alternative approach to control bacterial infections. J. Lasers Med. Sci..

[9] Jao Y., Ding S.-J., Chen C.-C. (2023). Antimicrobial photodynamic therapy for the treatment of oral infections: A systematic review. J. Dent. Sci..

[10] Dias L.D., Bertolo M.R.V., Alves F., de Faria C.M.G., Rodrigues M.Á.V., Lopes L.K.B.C., de Guzzi Plepis A.M., Mattoso L.H.C., Junior S.B., Bagnato V.S. (2022). Preparation and characterization of curcumin and pomegranate peel extract chitosan/gelatin-based films and their photoinactivation of bacteria. Mater. Today Commun..

[11] Dias L.D., Aguiar A.S.N., de Melo N.J., Inada N.M., Borges L.L., de Aquino G.L.B., Camargo A.J., Bagnato V.S., Napolitano H.B. (2023). Structural basis of antibacterial photodynamic action of curcumin against S. Aureus. Photodiagnosis Photodyn. Ther..

[12] Lima A.R., Dias L.D., Garbuio M., Inada N.M., Bagnato V.S. (2022). A Look at Photodynamic inactivation as a tool for pests and vector-borne diseases control. Laser Phys. Lett..

[13] Lima A.R., da Silva C.M., Caires C.S.A., Chaves H., Pancrácio A.S., de Arruda E.J., Caires A.R.L., Oliveira S.L. (2022). Photoinactivation of *Aedes aegypti* larvae using riboflavin as photosensitizer. Photodiagnosis Photodyn. Ther..

[14] Meier C.J., Hillyer J.F. (2024). Larvicidal Activity of the photosensitive insecticides, methylene blue and rose bengal, in *Aedes aegypti* and *Anopheles gambiae* Mosquitoes. Pest. Manag. Sci..

[15] Lima A.R., Silva C.M., da Silva L.M., Machulek A., de Souza A.P., de Oliveira K.T., Souza L.M., Inada N.M., Bagnato V.S., Oliveira S.L. (2022). Environmentally safe photodynamic control of *Aedes aegypti* using sunlight-activated synthetic curcumin: Photodegradation, aquatic ecotoxicity, and field trial. Molecules.

[16] Dias L.D., Blanco K.C., Mfouo-Tynga I.S., Inada N.M., Bagnato V.S. (2020). Curcumin as a photosensitizer: From molecular structure to recent advances in antimicrobial photodynamic therapy. J. Photochem. Photobiol. C Photochem. Rev..

[17] Garbuio M., Lima A.R., Silva K.J.S., De Souza M., Inada N.M., Dias L.D., Bagnato V.S. (2024). Influence of temperature combined with photodynamic inactivation on the development of *Aedes aegypti*. Photodiagnosis Photodyn. Ther..

[18] Perera K.D.C., Weragoda G.K., Haputhanthri R., Rodrigo S.K. (2021). Study of concentration dependent curcumin interaction with serum biomolecules using ATR-FTIR spectroscopy combined with principal component analysis (PCA) and partial least square regression (PLS-R). Vib. Spectrosc..

[19] Garbuio M., Dias L.D., de Souza L.M., Corrêa T.Q., Mezzacappo N.F., Blanco K.C., de Oliveira K.T., Inada N.M., Bagnato V.S. (2022). Formulations of curcumin and d-mannitol as a photolarvicide against *Aedes aegypti* larvae: Sublethal photolarvicidal action, toxicity, residual evaluation, and small-scale field trial. Photodiagnosis Photodyn. Ther..

[20] de Souza L.M., Venturini F.P., Inada N.M., Iermak I., Garbuio M., Mezzacappo N.F., de Oliveira K.T., Bagnato V.S. (2020). Curcumin in formulations against *Aedes aegypti*: Mode of action, photolarvicidal and ovicidal activity. Photodiagnosis Photodyn. Ther..

[21] Priyadarsini K. (2014). The Chemistry of curcumin: From extraction to therapeutic agent. Molecules.

[22] Adeluola A., Zulfiker A.H.M., Brazeau D., Amin A.R.M.R. (2021). Perspectives for synthetic curcumins in chemoprevention and treatment of cancer: An update with promising analogues. Eur. J. Pharmacol..

[23] Hegde M., Girisa S., BharathwajChetty B., Vishwa R., Kunnumakkara A.B. (2023). Curcumin formulations for better bioavailability: What we learned from clinical trials thus far?. ACS Omega.

[24] de Gomes M.G., Borges Filho C., Haas S.E. (2023). Technological Aspects and biological application of nanocapsules loaded with curcumin. Reviews in Studies in Natural Products Chemistry.

[25] Kazakova O., Lipkovska N., Barvinchenko V. (2022). Keto-enol tautomerism of curcumin in the preparation of nanobiocomposites with fumed silica. Spectrochim. Acta A Mol. Biomol. Spectrosc..

[26] Hartini N., Ponrasu T., Wu J.-J., Sriariyanun M., Cheng Y.-S. (2021). Microencapsulation of curcumin in crosslinked jelly fig pectin using vacuum spray drying technique for effective drug delivery. Polymers.

[27] Guo J., Li P., Kong L., Xu B. (2020). Microencapsulation of curcumin by spray drying and freeze drying. LWT.

[28] Lucas J., Ralaivao M., Estevinho B.N., Rocha F. (2020). A new approach for the microencapsulation of curcumin by a spray drying method, in order to value food products. Powder Technol..

[29] Manadas R., Pina M.E., Veiga F. (2002). Dissolution studies in vitro as a prognostic tool for oral absorption of modified release pharmaceutical dosage forms. Rev. Bras. De. Ciências Farm..

[30] Rodrigues L.C., Ribeiro A.P., Silva S.S., Reis R.L. (2024). Chitosan/virgin coconut oil-based emulsions doped with photosensitive curcumin loaded capsules: A functional carrier to topical treatment. Polymers.

[31] Silva L.S., Mar J.M., Azevedo S.G., Rabelo M.S., Bezerra J.A., Campelo P.H., Machado M.B., Trovati G., dos Santos A.L., da Fonseca Filho H.D. (2019). Encapsulation of *Piper aduncum* and *Piper hispidinervum* essential oils in gelatin nanoparticles: A possible sustainable control tool of *Aedes aegypti*, *Tetranychus urticae* and *Cerataphis lataniae*. J. Sci. Food Agric..

[32] World Health Organization (2005). World Health Organization Guidelines for Laboratory and Field Testing of Mosquito Larvicides.

[33] Vani J.M., Monreal M.T.F.D., Auharek S.A., Cunha-Laura A.L., de Arruda E.J., Lima A.R., da Silva C.M., Antoniolli-Silva A.C.M.B., de Lima D.P., Beatriz A. (2018). The mixture of cashew nut shell liquid and castor oil results in an efficient larvicide against *Aedes aegypti* that does not alter embryo-fetal development, reproductive performance or DNA integrity. PLoS ONE.

[34] Bhandari B.R., Datta N., Howes T. (1997). Problems associated with spray drying of sugar-rich foods. Dry. Technol..

[35] Cano-Higuita D.M., Malacrida C.R., Telis V.R.N. (2015). Stability of curcumin microencapsulated by spray and freeze drying in binary and ternary matrices of maltodextrin, gum arabic and modified starch. J. Food Process Preserv..

[36] Estevinho B.N., Damas A.M., Martins P., Rocha F. (2014). Microencapsulation of β-galactosidase with different biopolymers by a spray-drying process. Food Res. Int..

[37] Roshan Z., Haddadi-Asl V., Ahmadi H., Moussaei M. (2024). Curcumin-encapsulated poly (lactic-*Co*-glycolic acid) nanoparticles: A comparison of drug release kinetics from particles prepared via electrospray and nanoprecipitation. Macromol. Mater. Eng..

[38] Müller R.H. (1991). Colloidal Carriers for Controlled Drug Delivery and Targeting: Modification, Characterization And In Vivo Distribution.

[39] Chow S.F., Shi L., Ng W.W., Leung K.H.Y., Nagapudi K., Sun C.C., Chow A.H.L. (2014). Kinetic Entrapment of a hidden curcumin cocrystal with phloroglucinol. Cryst. Growth Des..

[40] Sasmita H.I., Tu W.C., Bong L.J., Neoh K.B. (2019). Effects of larval diets and temperature regimes on life history traits, energy reserves and temperature tolerance of male *Aedes aegypti* (Diptera: Culicidae): Optimizing rearing techniques for the sterile insect programmes. Parasit. Vectors.

[41] Chignell C.F., Bilskj P., Reszka K.J., Motten A.G., Sik R.H., Dahl T.A. (1994). Spectral and photochemical properties of curcumIN. Photochem. Photobiol..

[42] Ali Z., Saleem M., Atta B.M., Khan S.S., Hammad G. (2019). Determination of curcuminoid content in turmeric using fluorescence spectroscopy. Spectrochim. Acta A Mol. Biomol. Spectrosc..

[43] Nata I.F., Chen K.-J., Lee C.-K. (2014). Facile microencapsulation of curcumin in acetylated starch microparticles. J. Microencapsul..

[44] Chen Z., Xia Y., Liao S., Huang Y., Li Y., He Y., Tong Z., Li B. (2014). Thermal degradation kinetics study of curcumin with nonlinear methods. Food Chem..

[45] Liu R., Xu C., Cong X., Wu T., Song Y., Zhang M. (2017). Effects of oligomeric procyanidins on the retrogradation properties of maize starch with different amylose/amylopectin ratios. Food Chem..

[46] Lv X., Cao H., Li G., Zhu M., Ji W., Wang K., Zhang C., Su C., Ren W., Cai D. (2023). Spent yeast-derived 3d porous carbon skeleton as low-cost d-mannitol supporting material for medium temperature thermal energy storage. Materials.

[47] Parize A.L., Stulzer H.K., Laranjeira M.C.M., Brighente I.M.d.C., de Souza T.C.R. (2012). Evaluation of chitosan microparticles containing curcumin and crosslinked with sodium tripolyphosphate produced by spray drying. Quim. Nova.

[48] Qin Z., Liu H.-M., Cheng X.-C., Wang X.-D. (2019). Effect of drying pretreatment methods on structure and properties of pectins extracted from chinese quince fruit. Int. J. Biol. Macromol..

[49] Jiang Y., Xu Y., Li F., Li D., Huang Q. (2020). Pectin extracted from persimmon peel: A physicochemical characterization and emulsifying properties evaluation. Food Hydrocoll..

[50] Otegbayo B.O., Tanimola A.R., Ricci J., Gibert O. (2024). Thermal properties and dynamic rheological characterization of dioscorea starch gels. Gels.

[51] Liu X., Yu L., Liu H., Chen L., Li L. (2008). In situ thermal decomposition of starch with constant moisture in a sealed system. Polym. Degrad. Stab..

[52] Xu T., Chen Q., Huang G., Zhang Z., Gao X., Lu S. (2016). Preparation and thermal energy storage properties of d-mannitol/expanded graphite composite phase change material. Sol. Energy Mater. Sol. Cells.

[53] Opustilová K., Lapčíková B., Lapčík L., Gautam S., Valenta T., Li P. (2023). Physico-Chemical study of curcumin and its application in O/W/O multiple emulsion. Foods.

[54] Wani K.M., Uppaluri R.V.S. (2024). Efficacy of ionic gelation based encapsulation of bioactives from papaya leaf extract: Characterization and storage stability. Biomass Convers. Biorefin.

[55] Batool N., Sarfraz R.M., Mahmood A., Rehman U., Zaman M., Akbar S., Almasri D.M., Gad H.A. (2023). Development and evaluation of cellulose derivative and pectin based swellable ph responsive hydrogel network for controlled delivery of cytarabine. Gels.

[56] Godeck R., Kunzek H., Kabbert R. (2001). Thermal analysis of plant cell wall materials depending on the chemical structure and pre-treatment prior to drying. Eur. Food Res. Technol..

[57] Kaewnopparat N., Kaewnopparat S., Jangwang A., Maneenaun D., Chuchome T., Panichayupakaranant P. (2009). Increased solubility, dissolution and physicochemical studies of curcumin-polyvinylpyrrolidone K-30 solid dispersions. World Acad. Sci. Eng. Technol..

[58] Yang Y., Liu J., Hu A., Nie T., Cheng Z., Liu W. (2022). A critical review on engineering of d-mannitol crystals: Properties, applications, and polymorphic control. Crystals.

[59] Li B., Konecke S., Wegiel L.A., Taylor L.S., Edgar K.J. (2013). Both solubility and chemical stability of curcumin are enhanced by solid dispersion in cellulose derivative matrices. Carbohydr. Polym..

[60] Kaur R., Khullar P., Mahal A., Gupta A., Singh N., Ahluwalia G.K., Bakshi M.S. (2018). Keto-enol tautomerism of temperature and ph sensitive hydrated curcumin nanoparticles: Their role as nanoreactors and compatibility with blood cells. J. Agric. Food Chem..

[61] Angelini G., Pasc A., Gasbarri C. (2020). Curcumin in silver nanoparticles aqueous solution: Kinetics of keto-enol tautomerism and effects on AgNPs. Colloids Surf. A Physicochem. Eng. Asp..

[62] Kizil R., Irudayaraj J., Seetharaman K. (2002). Characterization of irradiated starches by using FT-raman and FTIR spectroscopy. J. Agric. Food Chem..

[63] Warren F.J., Gidley M.J., Flanagan B.M. (2016). Infrared spectroscopy as a tool to characterise starch ordered structure—A Joint FTIR–ATR, NMR, XRD and DSC study. Carbohydr. Polym..

[64] Bruni G., Berbenni V., Milanese C., Girella A., Cofrancesco P., Bellazzi G., Marini A. (2009). Physico-Chemical characterization of anhydrous d-mannitol. J. Therm. Anal. Calorim..

[65] Lin J., Huang J., Chen J., Lin Z., Ou Y., Yao L., Zhang X., Kang Q., Pan Y. (2016). Low cytotoxic d-mannitol isolated from the industrial wastewater of *Agaricus bisporus*. J. Food Nutr. Res..

[66] Sari A., Karaipekli A., Eroğlu R., Biçer A. (2013). Erythritol tetra myristate and erythritol tetra laurate as novel phase change materials for low temperature thermal energy storage. Energy Sources Part A Recovery Util. Environ. Eff..

[67] Tolun A., Sharifuzzaman M., Altintas Z. (2025). Electrospun nanofibers of curcumin/HP-Beta-CD/pullulan complex with enhanced solubility and controlled release in food and drug delivery applications. Int. J. Biol. Macromol..

[68] Liu C., Du W., Zhang L., Wang J. (2025). Natural synergy: Oleanolic acid-curcumin co-assembled nanoparticles combat osteoarthritis. Colloids Surf. B Biointerfaces.

[69] Sun X.Z., Williams G.R., Hou X.X., Zhu L.M. (2013). Electrospun curcumin-loaded fibers with potential biomedical applications. Carbohydr. Polym..

[70] Gnanasambandam R. (2000). Determination of pectin degree of esterification by diffuse reflectance fourier transform infrared spectroscopy. Food Chem..

[71] Santos E.E., Amaro R.C., Bustamante C.C.C., Guerra M.H.A., Soares L.C., Froes R.E.S. (2020). Extraction of pectin from agroindustrial residue with an ecofriendly solvent: Use of ftir and chemometrics to differentiate pectins according to degree of methyl esterification. Food Hydrocoll..

[72] Mezzacappo N.F., Souza L.M., Inada N.M., Dias L.D., Garbuio M., Venturini F.P., Corrêa T.Q., Moura L., Blanco K.C., Oliveira K.T. (2021). Curcumin/D–Mannitol as Photolarvicide: Induced Delay in Larval Development Time, Changes in Sex Ratio and Reduced Longevity of Aedes Aegypti. Reviews in Pest Management Science.

[73] Melo-Santos M.A.V., Varjal-Melo J.J.M., Araújo A.P., Gomes T.C.S., Paiva M.H.S., Regis L.N., Furtado A.F., Magalhaes T., Macoris M.L.G., Andrighetti M.T.M. (2010). Resistance to the organophosphate temephos: Mechanisms, evolution and Reversion in an *Aedes aegypti* Laboratory Strain from Brazil. Acta Trop..

[74] Cesar F., Betim M., Oliveira C.F.D., Montrucchio D.P., Miguel O.G., Miguel M.D., Bello J., Maurer B., de Fátima Gaspari Dias J., Dias G. (2021). Short communication preliminary evaluation of the larvicidal activity of extracts and fractions from Ocotea Nutans (Nees) Mez against *Aedes aegypti*. Rev. Da Soc. Bras. De Med. Trop..

[75] Eukubay A., Getu E., Debebe E., Hadis M. (2020). Larvicidal potential of some plant extracts against *Anopheles arabiensis* patton (diptera: Culicidae). Int. J. Trop. Insect Sci..

[76] de Carvalho G.H.F., de Andrade M.A., de Araújo C.N., Santos M.L., de Castro N.A., Charneau S., Monnerat R., de Santana J.M., Bastos I.M.D. (2019). Larvicidal and pupicidal activities of eco-friendly phenolic lipid products from anacardium occidentale nutshell against arbovirus vectors. Environ. Sci. Pollut. Res..

[77] Lima A.R., Silva C.M., Caires C.S.A., Prado E.D., Rocha L.R.P., Cabrini I., Arruda E.J., Oliveira S.L., Caires A.R.L. (2018). Evaluation of eosin-methylene blue as a photosensitizer for larval control of *Aedes aegypti* by a photodynamic process. Insects.

[78] de Souza L.M., Inada N.M., Venturini F.P., Carmona-Vargas C.C., Pratavieira S., de Oliveira K.T., Kurachi C., Bagnato V.S. (2019). Photolarvicidal effect of curcuminoids from curcuma longa linn. against *Aedes aegypti* Larvae. J. Asia Pac. Entomol..

[79] Wang Y.-J., Pan M.-H., Cheng A.-L., Lin L.-I., Ho Y.-S., Hsieh C.-Y., Lin J.-K. (1997). Stability of curcumin in buffer solutions and characterization of its degradation products. J. Pharm. Biomed. Anal..

[80] Boudko D.Y., Moroz L.L., Harvey W.R., Linser P.J. (2001). Alkalinization by chloride/bicarbonate pathway in larval mosquito midgut. Proc. Natl. Acad. Sci. USA.

[81] Clements A.N. (1963). The Physiology of Mosquitoes.

[82] Paiva F.L.P., Silva M.V.C., Mendonça A.L.F., Araújo C.S., Sallum L.O., Aguiar A.S.N., Lima A.R., Napolitano H.B., Calvete M.J.F., Dias L.D. (2024). Photocatalytic Degradation of Ciprofloxacin: A Combined Experimental and Theoretical Study Using Curcumin and Hydrogen Peroxide. Separations.

[83] Antoniazzi C., Castro E.G., Anaissi F.J. (2018). Zirconium oxide and iron zirconate obtained from citrus pectin and nitrates applied in the photo-fenton-like process. Orbital Electron. J. Chem..

[84] Korsmeyer R., Peppas N. (1983). Macromolecular and modeling aspects of swelling controlled systems. Control. Release Deliv. Syst..

[85] Maderuelo C., Zarzuelo A., Lanao J.M. (2011). Critical factors in the release of drugs from sustained release hydrophilic matrices. J. Control. Release.

[86] Siepmann J., Siepmann F. (2013). Mathematical modeling of drug dissolution. Int. J. Pharm..

[87] Vinceković M., Jurić S., Đermić E., Topolovec-Pintarić S. (2017). Kinetics and mechanisms of chemical and biological agents release from biopolymeric microcapsules. J. Agric. Food Chem..

[88] Boyapally H., Nukala R.K., Bhujbal P., Douroumis D. (2010). Controlled release from directly compressible theophylline buccal tablets. Colloids Surf. B Biointerfaces.

[89] Lopalco A., Marinaro W.A., Day V.W., Stella V.J. (2017). Isolation, solubility, and characterization of d-mannitol esters of 4-methoxybenzeneboronic acid. J. Pharm. Sci..

