# Supplementary material for: Curcumin Microcapsule Formulations for Prolong Persistence in the Photodynamic Inactivation of Aedes aegypti Larvae
Source: Pharmaceutics. 2025 Apr 9;17(4):496. doi: 10.3390/pharmaceutics17040496 (PMC12030088; doi:10.3390/pharmaceutics17040496)
Supplement: Supplementary file 1 [file pharmaceutics-17-00496-s001.zip › pharmaceutics-3533447-supplementary.pdf]

SUPPLEMENTARY MATERIAL

Curcumin microcapsule formulations for prolong persistence in the photodynamic inactivation of *Aedes aegypti* larvae

Matheus Garbuio[1,2,3,] Larissa Marila de Souza[1], Lucas Danilo Dias[4], Jean Carlos Ferreira Machado[5], Natalia Mayumi Inada[1], Hernane da Silva Barud[5], Edgar Aparecido Sanches[6], Francisco Eduardo Gontijo Guimarães[1], Ana Paula da Silva[1], Alessandra Ramos Lima[1,2], Vanderlei Salvador Bagnato[1,2,7]

[1]São Carlos Institute of Physics (IFSC), University of São Paulo (USP), São Carlos, SP, 13566-590, Brazil
[2]Environmental Biophotonics Laboratory, São Carlos Institute of Physics, University of São Paulo, São Carlos, SP, 13566-590 Brazil
[3]PPG Biotec, Federal University of São Carlos, São Carlos, 13565-905, SP, Brazil
[4]Laboratório de Novos Materiais, Universidade Evangélica de Goiás, Anápolis, GO, 75083-515, Brazil
[5]Biopolymers and Biomaterials Laboratory (BioPolMat), University of Araraquara - UNIARA, Araraquara, S P14801-320, Brazil
[6]Laboratory of Nanostructured Polymers (NANOPOL), Federal University of Amazonas (UFAM), Manaus, AM, 69080-005, Brazil
[7]Department of Biomedical Engineering, Texas A&M University, College Station, TX, 77843, USA

*Corresponding author at: lucasdanillodias@gmail.com

1 Analytical curve construction

The analytical curve was constructed in triplicate for curcumin, the maximum absorption value at the wavelength of 430±2 nm used to obtain the linear regression fitting function. The calibration curve for free curcumin was prepared in a stock solution of 99% ethanol (EtOH) and 1% dimethyl sulfoxide (DMSO) at a concentration of 500 mg/L. Subsequently, dilution to a concentration of 20 mg/L was performed in a ratio of distilled water and EtOH (50:50)%, the calibration curve was obtained in the range between 0.0-7.0 mg/L and a linear correlation coefficient R^2 =0.99993, represented by the equation y = -0.00286 + 155.08293x (Figure S1). The limit of detection and limit of quantification are 0.00214 mg/L and 0.007 mg/L, respectively.

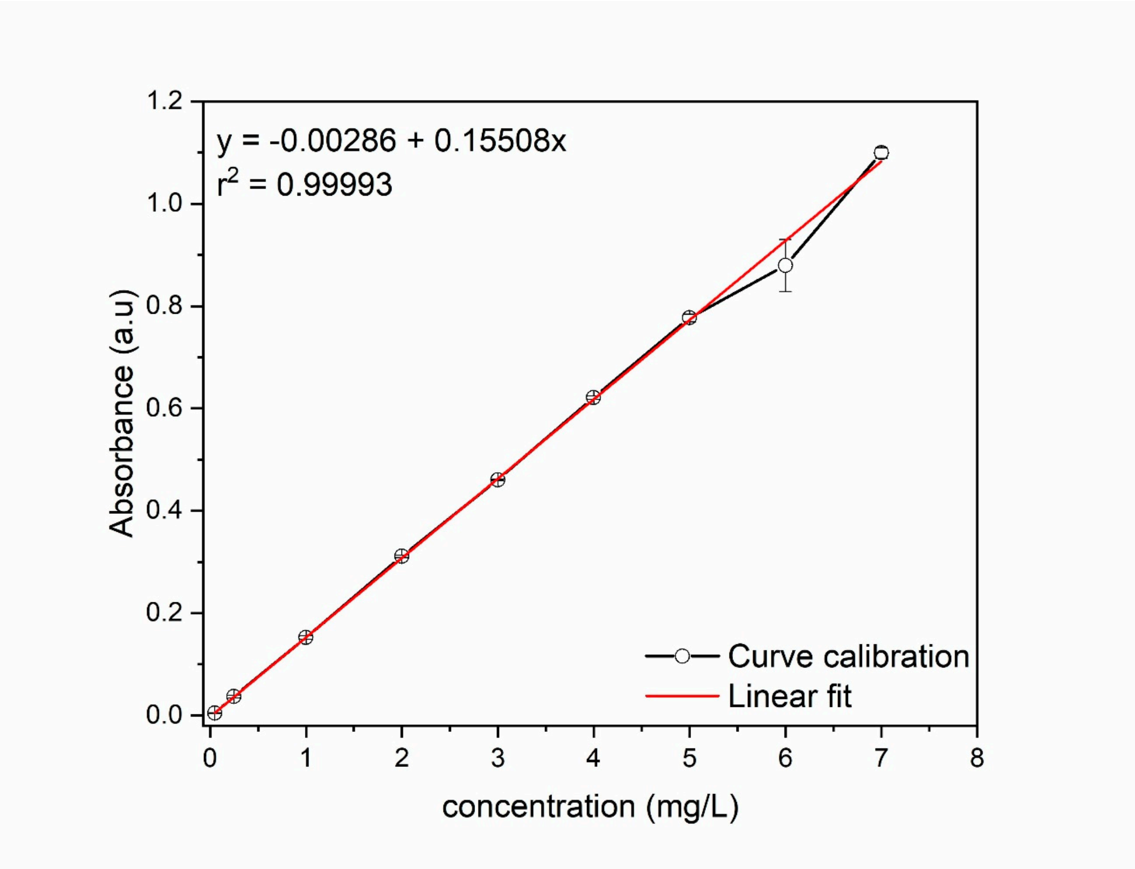

Figure S1. Curve calibration curcumin, concentration 0.0-7.0 mg/L and a linear correlation coefficient R^2 0.99993.

2 Extracting curcumin from microcapsules

The extraction of curcumin from the microcapsules was performed with 99% EtOH as the extracting solvent and detection by ultraviolet-visible absorption spectroscopy (UV-Vis) Cary 50 Bio, scanning 200-800 nm, using a quartz cuvette. To optimize the extraction process, the variables extraction time, types of filter paper, volume of extracting solvent and samples were evaluated (Data not shown). The best optimized conditions were chosen to perform the extraction of curcumin. The mass of a 200 mg

tablet was added to 1000 mL of distilled water (H_2O) and stirred for 30 min, in triplicate. Then, 5.00 mL aliquots of the sample were collected and 5.00 mL of 99% EtOH were added, maintaining the ratio of 50:50% (H_2O:EtOH) with constant stirring for 20 min. Subsequently, filtration was performed using 12.5 cm black band quantitative filter paper (weight 85 g/m^2 and thickness 0.20 mm). Then, 2.5 mL of the sample was transferred to a 10 mL volumetric flask and adjusted with a 50:50 ratio of H_2O:EtOH. Then, UV-Vis absorption analyses were performed and the concentration was further determined using the analytical curve (Figure S1).
